# Physiological mechanisms of ABA-induced salinity tolerance in leaves and roots of rice

**DOI:** 10.1038/s41598-022-11408-0

**Published:** 2022-05-17

**Authors:** Guanjie Chen, Dianfeng Zheng, Naijie Feng, Hang Zhou, Dewei Mu, Liming Zhao, Xuefeng Shen, Gangshun Rao, Fengyan Meng, Anqi Huang

**Affiliations:** 1 College of Coastal Agricultural Sciences, Guangdong Ocean University, Zhanjiang, 524088 China; 2 Shenzhen Research Institute of Guangdong Ocean University, Shenzhen, 518108 China; 3 South China Center of National Salt-Alkali Tolerant Rice Technology Innovation Center, Zhanjiang, 524088 Guangdong China; 4 School of Tropical Crops, Hainan University, Haikou, 570228 China

**Keywords:** Biochemistry, Physiology

## Abstract

Abscisic acid (ABA) plays a crucial role in response to abiotic stress as important small molecules in regulating metabolism. This study aimed to evaluate the ability of foliar spraying ABA to regulate growth quality at rice seedling stage under salt stress. Results demonstrated that salt stress strongly reduced all the growth parameters of two rice seedlings (‘Chaoyouqianhao’ and ‘Huanghuazhan’), caused prominent decrease in the levels of photosynthetic pigments (mainly in Huanghuazhan), photosynthesis and fluorescence parameters. Salinity treatment increased the concentration of malondialdehyde (MDA) and hydrogen peroxide (H_2_O_2_) in roots, whereas significant decreased H_2_O_2_ was found in leaves of Huanghuazhan. Additionally, salinity triggered high Na^+^ content particularly in leaves and enhanced catalase (CAT) activities, ascorbate peroxidase (APX) and peroxidase (POD) activities of the two rice seedlings. Nevertheless, salinity-induced increased root ascorbic acid (AsA) and glutathione (GSH) levels while decreased in leaves, which depended on treatment time. Conversely, ABA application partially or completely mitigated salinity toxicity on the seedlings. ABA could reverse most of the changed physiological parameters triggered by salt stress. Specially, ABA treatment improved antioxidant enzyme levels and significantly reduced the Na^+^ content of two varieties as well as increased the K^+^, Mg^2+^ and Ca^2+^ content in leaves and roots. ABA treatment increased the hormone contents of 1-aminocclopropane carboxylic acid (ACC), trans-zeatin (TZ), N6-isopentyladenosine (IPA), Indole-3-acetic acid (IAA), and ABA in leaves of two rice varieties under salt stress. It is suggested that ABA was beneficial to protect membrane lipid peroxidation, the modulation of antioxidant defense systems and endogenous hormonal balance with imposition to salt stress.

## Introduction

Soil area affected by salinity is increasing to global warming, high temperatures, and rising sea levels. According to statistics, high soil salinity turns about 6% (800 million ha) of worldwide fertile land into unarable land^1^. Naturally, salt stress is the most important stress factor among them that not only leads to an imbalance in the Earth's climatic conditions, but also has destructive effects on plant growth and physiological processes. It is estimated that salt stress reduces crop production in about 20% of irrigated land worldwide, resulting in annual losses of as much as $12 billion worldwide. On the other hand, 50% of the arable land will be lost due to salinity by the mid-twenty-first century^2–4^.

Salinity in the soil adversely affects root growth and reduces the ability of plants to absorb water and other nutrients from the soil, thus causing delayed plants growth even ultimately lead to plants death. Actually, soil salinity causes iotoxicity, osmotic stress and nutrients deficit in plants. In addition, salt stress leads to membrane damage and stomatal closure, which results in reduced carbon dioxide uptake, hydrolase activity, and increased lipid peroxidation levels, which may stimulate a large number of formation of ROS, such as O_2_^−^, H_2_O_2_, and OH^−^. These molecules could trigger oxidative damage to cell membranes, proteins, DNA, and lipids, simultaneously accompanied by the accumulation of MDA, which further disrupted the various biochemical and metabolic processes in plants^5–7^. Hence, plants must evolved different strategies to mitigate the harmful effects of salt stress and to protect themselves from the harmful effects of ROS. Antioxidant defense system as an effective strategy, including enzymatic antioxidants and non-enzymatic antioxidants. The former mainly includes SOD, POD, CAT, APX, GR and the latter includes some low-molecular-weight compounds, such as carotenoid, flavonoids, AsA, or GSH, which involved in different metabolic processes in cells and acting as cofactors for different enzymes, ultimately affecting plant growth and development^8^. Admittedly, antioxidant defense system could be effectively eliminated ROS and maintain appropriate equilibrium within the cells^9^. In addition, intracellular ion homeostasis (mainly K^+^/Na^+^), synthesis and accumulation of compatible solutes such as proline and betaine, are two equally important strategies, which are fundamental to the basis of cell physiology and contribute to improve salt tolerance.

Rice (*Oryza Sativa L.*) is an important food source for more than half of the world's population. However, as a salt-sensitive crop, the harmful effect of salt stress to rice has become a core problem of global rice production. It was demonstrated that the degree of salt damage to rice depends on the duration of salt treatment and the growth stage of plants^10^. Rice may impose salt stress during the germination, seedling or reproductive growth stages, and is considered to be most susceptible to salt stress during 2–3 leaf stage^11^. Salinity triggered a variety of unfavorable impacts on rice, including plant phenotype, biochemical, physiological, cellular, and molecular levels, and ultimately affected the productivity. Hence, how to promote the normal growth and development of rice under high soil salinity and improve the productivity has become an urgent problem at present. Studies of crop salt tolerance mechanism have showed that using chemical regulation is one of the effective measures to improve crop salt tolerance^12^.

ABA is one of the most important hormones in regulating antioxidant defense systems under environmental stress^13^. Studies have shown that exogenous ABA can improve the crop tolerance in response to drought^14^, salinity^15^, heavy metal^16^, cold^17^ and heat^18^. For example, ABA application improved the survival and growth potential of rice under salt stress^19,20^. It is demonstrated that ABA improved tolerance to alkaline stress by initiating antioxidant defense systems in roots of rice seedling and upregulating genes related in tolerance^21^. In addition, since ABA plays an important role in promoting efflux of K^+^, Cl^−^ and other ions, plants could synthesize a large amount of ABA after being stimulated by salinity, caused stomatal closure and played a certain protective effect. Besides, ABA could reduce stomatal conductance, water consumption, wilting and transpiration; on the other hand, it is conducive to maintain low Na^+^/K^+^ as well as enhance the activity of protective enzymes, ultimately keep an intact cell membrane structure and reduce the salinity-induced damage^22^.

At present, there are many studies on growth characteristic, oxidative stress and antioxidant defense mechanisms in response to salt stress on leaf or root system^23–27^. However, Few reports have been made comparing differences in antioxidant systems in leaves or roots. Previous study showed that leaf tissue suffered greater oxidative stress-mediated damage than root tissue of lentils under salt stress^28^. Additionally, exogenous ABA participated in regulating the distribution of auxin so that enhanced the lateral root growth of maize under salt stress^29^. Liu et al. showed that proper concentration of exogenous ABA could enhance tolerance of rice roots to simulated acid rain stress by promoting nutrients uptake and balancing endogenous hormones^30^. Zhang et al. demonstrated that ABA application improved leaf drought resistance by improving relative water content and ABA accumulation as well as reducing osmotic stress damage to leaves (low MDA content)^31^. A research from Leng et al. manifested that there were differences in the physiological and biochemical response patterns of roots, stems and leaves to Cd and ABA in mung bean seedlings^16^. It is not only important to study morphological and physiological changes in plants, but also to analyze the comparative response to different cultivar species. Particularly, the differential responses of plant roots and leaves to abiotic stresses and ABA application remain unclear. Thus, this study provided some insights into the phenotyping and physiological mechanisms of leaves and roots of two rice varieties in response to ABA application during five salinity treatment time. Specially, growth performance, photosynthesis, fluorescence, mineral homeostasis, oxidative stress, antioxidant defend systems and endogenous hormone homeostasis was assessed.

## Result

### Effect of different concentrations of ABA on rice seedling growth under salt stress

**Figure 1:**
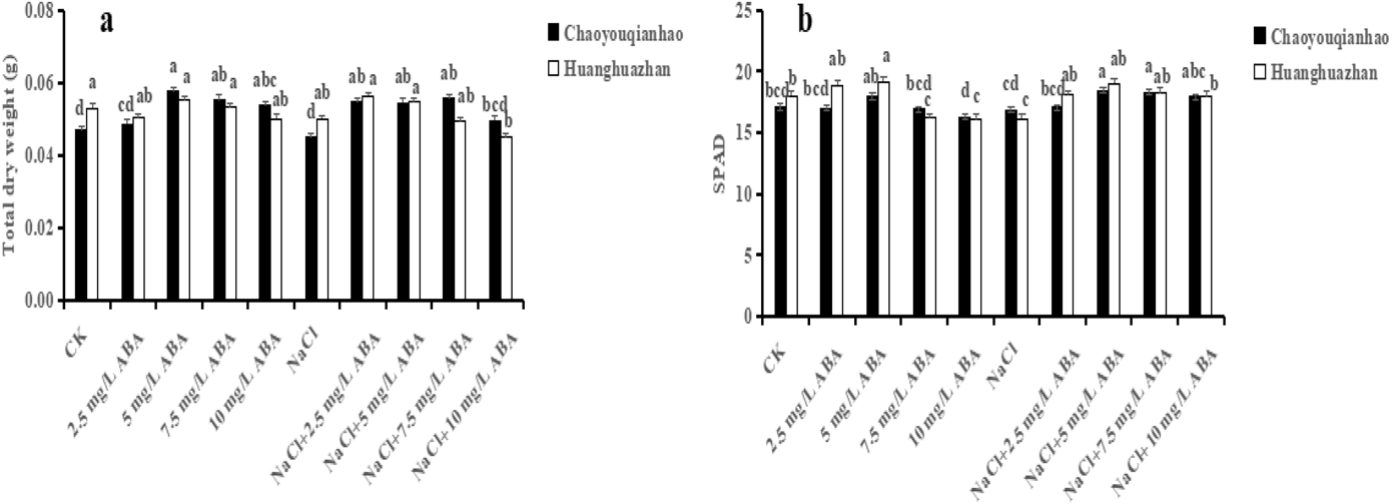
Effect of ABA with leaf sprayed on total dry weight (**a**) and SPAD (**b**) of two rice cultivar seedlings under salt stress. Values are shown as the mean ± standard and derived from four replicates (n = 4). Bars indicate standard errors. Different letters above the bars indicate significant differences at the five percent significant level according to Duncan's multiple range tests.

As shown in Fig. 1a, different concentrations (2.5 mg/L, 5.0 mg/L, 7.5 mg/L, 10.0 mg/L) of exogenous ABA showed an increased trend under normal condition (non-saline) in terms of total dry weight, in comparison with the control. Moreover, the peak value of total dry weight was found under 5.0 mg/L ABA among the four ABA concentrations, which increased by 22.77% when compared to the control, reached to a significant level (p < 0.05). An inhibitory effect was observed in terms of total dry weight underlying salinity treatment in contrast to the control, which slightly decreased by 4.28% and 5.55% in Chaoyouqianhao and Huanghuazhan, respectively. Notably, the changed trend was reversed by 2.5 and 5.0 mg/L ABA in both of the rice cultivars, which was enhanced by 21.93%, 21.72% in Chaoyouqianhao and 12.63%, 8.98% in Huanghuazhan, respectively.

Similarly, application of 5.0 mg/L ABA in SPAD displayed the optimal increment whenever in the 5.0 mg/L ABA alone treatment (non-saline) or NaCl + 5 mg/L ABA treatment (Fig. 1b). Therefore, 5.0 mg/L ABA was considered an appropriate concentration for foliar application in subsequent experiments.

### Effects of exogenous ABA on the shoot morphological parameters under salt stress

Salinity treatment exhibited different degrees of reduction in all shoot morphological parameters during the treatment time course (Table 1). In 2–4 days, plant height shown a remarkable reduction in Q2 (on average by 8.69%) and Z2 (on average by 10.53%) when compared to Q0 and Z0, respectively; these trend became stronger in 6–10 days, which shown an average 16.26% decrease in Q2 and 11.44% decrease in Z2, respectively. When compared with salinity treatment alone (Q2 and Z2), ABA treatment restored the decreased plant height caused by salinity, which resulted in a slight increase in Q3 (on average by 1.69%) and Z3 (on average by 1.53%), respectively, from 6 to 10 days.

**Table 1:** Effects of exogenous ABA on the shoot morphological parameters of five treatment time course in the presence of salinity.

Shoot morphological index	Treatments	Duration of salt stress (days)				
		2	4	6	8	10
Plant height (cm)	Q_0_	23.68 ± 0.39a	26.67 ± 0.32a	29.39 ± 0.32a	31.15 ± 0.30a	31.65 ± 0.29a
	Q_1_	21.91 ± 0.29b	25.05 ± 0.31b	25.33 ± 0.27b	25.52 ± 0.24bc	27.94 ± 0.41b
	Q_2_	21.14 ± 0.37b	24.90 ± 0.48b	25.15 ± 0.60b	25.98 ± 0.37b	26.03 ± 0.38c
	Q_3_	22.04 ± 0.38b	25.27 ± 0.21b	25.35 ± 0.25b	24.89 ± 0.16c	26.70 ± 0.44c
	Z_0_	26.03 ± 0.23b	29.68 ± 0.58a	31.23 ± 0.38a	30.35 ± 0.30a	31.08 ± 0.38a
	Z_1_	27.22 ± 0.24a	28.53 ± 0.35ab	28.70 ± 0.34b	29.66 ± 0.55a	31.36 ± 0.45a
	Z_2_	23.05 ± 0.74d	26.83 ± 0.39c	27.14 ± 0.36c	27.42 ± 0.28b	27.48 ± 0.38b
	Z_3_	25.04 ± 0.22c	27.27 ± 0.37bc	27.33 ± 0.37c	27.79 ± 0.25b	28.18 ± 0.69b
Stem diameter (mm)	Q_0_	1.99 ± 0.02ab	2.92 ± 0.07a	3.28 ± 0.06a	3.38 ± 0.07a	3.49 ± 0.13a
	Q_1_	2.01 ± 0.02ab	2.69 ± 0.06b	2.71 ± 0.04bc	3.29 ± 0.05ab	3.38 ± 0.06ab
	Q_2_	1.93 ± 0.03b	2.86 ± 0.06ab	2.68 ± 0.07c	3.11 ± 0.08b	3.13 ± 0.11b
	Q_3_	2.03 ± 0.03a	2.98 ± 0.06a	2.89 ± 0.06b	3.27 ± 0.08ab	3.32 ± 0.10ab
	Z_0_	2.14 ± 0.06a	2.84 ± 0.05a	2.60 ± 0.05a	3.37 ± 0.08a	3.43 ± 0.10a
	Z_1_	2.11 ± 0.07a	2.81 ± 0.10a	2.66 ± 0.08a	3.26 ± 0.05a	3.46 ± 0.06a
	Z_2_	1.90 ± 0.05b	2.51 ± 0.09b	2.53 ± 0.08a	2.69 ± 0.08b	2.95 ± 0.09b
	Z_3_	2.03 ± 0.07ab	2.83 ± 0.09a	2.67 ± 0.07a	3.29 ± 0.09a	3.29 ± 0.08a
Leaf length (mm)	Q_0_	117.33 ± 3.23ab	240.89 ± 6.47a	230.40 ± 8.30a	225.67 ± 6.62a	167.13 ± 5.36a
	Q_1_	110.50 ± 2.71b	184.13 ± 8.60b	152.30 ± 4.51b	237.00 ± 4.58a	158.10 ± 7.25ab
	Q_2_	116.45 ± 3.47ab	199.20 ± 7.95b	221.33 ± 5.78a	228.88 ± 7.21a	148.20 ± 4.68b
	Q_3_	127.40 ± 4.16a	190.50 ± 9.61b	226.75 ± 5.64a	236.70 ± 6.26a	160.30 ± 4.99ab
	Z_0_	108.64 ± 3.56b	163.88 ± 5.59a	166.60 ± 6.35b	214.71 ± 10.20a	144.25 ± 7.97a
	Z_1_	111.05 ± 2.31b	165.38 ± 8.95a	220.00 ± 11.72a	225.50 ± 14.56a	152.40 ± 6.59a
	Z_2_	101.67 ± 5.85b	156.50 ± 15.09a	160.00 ± 9.70b	165.33 ± 9.78b	101.38 ± 5.63b
	Z_3_	162.80 ± 16.11a	174.00 ± 8.16a	182.67 ± 8.01b	201.67 ± 11.64a	145.30 ± 7.31a
Leaf area (mm^2^)	Q_0_	354.18 ± 15.78b	812.53 ± 32.58a	590.76 ± 20.75ab	537.22 ± 28.81b	519.03 ± 58.24b
	Q_1_	391.68 ± 18.18b	759.70 ± 15.67a	512.69 ± 32.00c	585.81 ± 27.87b	450.26 ± 37.04b
	Q_2_	343.76 ± 22.20b	785.36 ± 30.41a	544.78 ± 19.65bc	541.04 ± 28.02b	527.14 ± 45.86b
	Q_3_	516.75 ± 13.69a	804.70 ± 15.12a	641.71 ± 24.24a	679.68 ± 25.80a	762.19 ± 18.41a
	Z_0_	270.11 ± 12.04b	431.37 ± 21.95a	384.21 ± 30.69a	338.56 ± 24.36b	180.63 ± 11.94b
	Z_1_	319.12 ± 17.56a	577.13 ± 27.69b	369.91 ± 38.19a	417.58 ± 29.39a	198.28 ± 10.02b
	Z_2_	292.42 ± 2.65ab	422.95 ± 63.31b	254.74 ± 12.57b	203.23 ± 19.63c	118.42 ± 12.96c
	Z_3_	309.42 ± 8.88ab	570.83 ± 30.20a	331.33 ± 19.70ab	436.36 ± 21.79a	347.08 ± 26.29a

Q_0_ treatment of water, Q_1_ treatment with ABA, Q_2_ treatment with NaCl, Q_3_ treatment with ABA + NaCl in Chaoyouqianhao. Z_0_ treatment of water, Z_1_ treatment with ABA, Z_2_ treatment with NaCl, Z_3_ treatment with ABA + NaCl in Huanghuazhan. Datas are mean ± standard error of at least four replicates. Within each column, different letters indicate significant difference at the five percent significant level according to Duncan's multiple range tests.

As shown in Table 1, salt stress prominently decreased the stem diameter in Q2 (on average by 12.20%) and Z2 (on average by 12.29%) compared to their respective control, in 6–10 days; however, these changed trend was reversed by application of ABA, which showed an average 6.35% increase in Q3 and 13.12% increase in Z3, respectively.

In comparison with Q0, salt stress treatment resulted in a slight reduction in leaf length and leaf area in Q2 during the treatment time course (Table 1). Notably, ABA + NaCl treatment (Q3) significantly increased the leaf area (on average by 34.58%) whereas a minor difference was found in leaf length during 2–10 days. Slight reduction was found in leaf length in Z2 when compared with Z0, from 2 to 6 days (Table 1), however, this trend became obvious from 8 to 10 days, which was decreased by 26.36% on average; notably, ABA + NaCl treatment (Z3) significantly increased the leaf length by 32.65% on average when compared to Z2 (8–10 days). Similarly, obvious reduction (on average by 36.04%) in leaf area was obtained in the presence of salinity (Z2) in 6–10 days, it was worth noting that ABA application strongly increased the leaf area by 112.62% on average in Z3 (6–10 days).

### Effects of exogenous ABA on the root morphological parameters under salt stress

In present study, we observed that salinity treatment (Q2 and Z2) exhibited an remarkable reduction in RL during treatment time course in comparison with Q0 and Z0, respectively; which decreased by 19.58% in Q2 and 24.84% on average in Z2, respectively (Table 2). In comparison with Q2, ABA restored the decreased RL caused by salinity in Q3 during 2–4 days (increased on average by 30.45%) and 8 days after salt stress (increased by 19.37%), respectively. Likewise, ABA treatment strongly increased RL in Z3 during 4–10 days (on average by 37.68%) when compared with Z2.

**Table 2:** Effects of exogenous ABA on the root morphological parameters of five treatment time course in the presence of salinity.

Root morphology indexes	Treatments	Duration of salt stress (days)				
		2	4	6	8	10
RL (cm)	Q_0_	1527.297 ± 72.284b	1865.936 ± 57.217a	1887.065 ± 93.148a	1913.791 ± 42.822a	2237.531 ± 43.270a
	Q_1_	2061.009 ± 68.806a	1722.481 ± 87.213ab	1918.364 ± 71.524a	1905.424 ± 49.963a	1740.499 ± 23.042c
	Q_2_	992.968 ± 45.561c	1330.461 ± 37.333c	1640.730 ± 60.905b	1664.085 ± 43.595b	2055.878 ± 42.923b
	Q_3_	1428.053 ± 33.056b	1557.651 ± 62.433b	1509.628 ± 55.048b	1986.373 ± 41.022a	2078.838 ± 54.698b
	Z_0_	1244.785 ± 50.296b	1263.332 ± 55.976c	1608.817 ± 162.487b	1687.928 ± 78.604a	1923.892 ± 64.187b
	Z_1_	1669.422 ± 80.943a	1665.656 ± 35.923a	2025.025 ± 57.653a	1703.851 ± 50.350a	1860.486 ± 42.543b
	Z_2_	1004.936 ± 32.768c	1103.805 ± 60.720d	997.324 ± 36.160d	1279.446 ± 40.500c	1345.150 ± 50.764c
	Z_3_	1110.820 ± 84.072bc	1492.566 ± 26.671b	1334.720 ± 77.917c	1482.172 ± 47.045b	2230.447 ± 74.359a
RSA (cm^2^)	Q_0_	77.443 ± 6.093b	107.159 ± 4.492a	104.072 ± 5.089a	191.917 ± 6.039b	238.443 ± 8.565a
	Q_1_	105.594 ± 4.721a	93.546 ± 6.702b	115.083 ± 6.397a	212.187 ± 7.484a	174.394 ± 4.696b
	Q_2_	60.895 ± 3.140c	78.845 ± 2.332c	116.837 ± 5.083a	176.216 ± 7.137b	227.161 ± 9.653a
	Q_3_	71.728 ± 2.115b	98.103 ± 4.038ab	104.744 ± 3.005a	226.006 ± 5.454a	247.840 ± 7.546a
	Z_0_	59.470 ± 2.358b	66.816 ± 1.894bc	84.653 ± 11.743b	154.105 ± 2.585b	209.023 ± 6.303b
	Z_1_	79.943 ± 5.110a	78.682 ± 4.724ab	117.393 ± 4.428a	185.708 ± 7.725a	218.185 ± 6.270b
	Z_2_	48.672 ± 1.968c	63.917 ± 5.740c	56.266 ± 1.872c	132.028 ± 3.927c	136.310 ± 4.974c
	Z_3_	51.533 ± 4.111bc	80.546 ± 1.971a	79.469 ± 5.121b	167.336 ± 6.181b	244.669 ± 9.127a
RV (cm^3^)	Q_0_	0.824 ± 0.116b	1.256 ± 0.081ab	1.101 ± 0.034b	3.088 ± 0.117c	3.991 ± 0.151b
	Q_1_	1.063 ± 0.063a	0.996 ± 0.091b	1.308 ± 0.054b	3.626 ± 0.131bc	2.637 ± 0.090c
	Q_2_	0.596 ± 0.086b	1.239 ± 0.059ab	1.858 ± 0..071a	3.999 ± 0.187ab	4.114 ± 0.275b
	Q_3_	0.694 ± 0.048b	1.518 ± 0.164a	1.971 ± 0.121a	4.654 ± 0.265a	4.870 ± 0.215a
	Z_0_	0.599 ± 0.051b	1.313 ± 0.163ab	1.334 ± 0.338ab	2.763 ± 0.228b	3.359 ± 0.122c
	Z_1_	0.810 ± 0.081a	1.339 ± 0.151ab	1.658 ± 0.071a	3.511 ± 0.213a	3.737 ± 0.122b
	Z_2_	0.508 ± 0.030b	0.887 ± 0.085b	0.983 ± 0.060b	2.335 ± 0.087b	1.986 ± 0.111d
	Z_3_	0.517 ± 0.036b	1.409 ± 0.120a	1.175 ± 0.154b	3.580 ± 0.126a	4.156 ± 0.116a
RAD (cm)	Q_0_	0.151 ± 0.006b	0.171 ± 0.002ab	0.170 ± 0.003b	0.305 ± 0.005b	0.324 ± 0.004b
	Q_1_	0.154 ± 0.003ab	0.163 ± 0.004b	0.170 ± 0.002b	0.332 ± 0.005a	0.309 ± 0.004c
	Q_2_	0.169 ± 0.008a	0.174 ± 0.002a	0.194 ± 0.004a	0.316 ± 0.004b	0.337 ± 0.005a
	Q_3_	0.152 ± 0.002b	0.178 ± 0.004a	0.195 ± 0.004a	0.332 ± 0.005a	0.347 ± 0.003a
	Z_0_	0.144 ± 0.002b	0.143 ± 0.003a	0.151 ± 0.004c	0.317 ± 0.003ab	0.321 ± 0.004a
	Z_1_	0.152 ± 0.003a	0.141 ± 0.003a	0.166 ± 0.003ab	0.326 ± 0.005a	0.326 ± 0.007a
	Z_2_	0.146 ± 0.002ab	0.151 ± 0.006a	0.158 ± 0.002bc	0.305 ± 0.006b	0.294 ± 0.004b
	Z_3_	0.139 ± 0.002b	0.144 ± 0.003a	0.169 ± 0.002a	0.329 ± 0.004a	0.328 ± 0.003a

RL indicates root length, RSA indicates root surface area, RV indicates root total volume, RAD indicated root average diameter. Q_0_ treatment of water, Q_1_ treatment with ABA, Q_2_ treatment with NaCl, Q_3_ treatment with ABA + NaCl in Chaoyouqianhao. Z_0_ treatment of water, Z_1_ treatment with ABA, Z_2_ treatment with NaCl, Z_3_ treatment with ABA + NaCl in Huanghuazhan. Values are mean ± standard error of four replicates (n = 4). Within each column, different letters indicate significant difference at the five percent significant level according to Duncan's multiple range tests.

As shown in Table 2, the RSA exhibited an obvious decline from 2 to 4 days (on average by 23.90%) whereas a slight decrease from 8 to 10 days (on average by 6.46%) in Q2 when relative to Q0, which were reversed by ABA from 2 to 4 days and 8 days after salt stress in Q3, elevated by 21.11% on average and 28.26%, respectively. However, salinity alone treatment significantly inhibited the RSA during the treatment time course (2–10 days) in Z2 when relative to Z0, which decreased by 25.20% on average. Notably, ABA + NaCl treatment (Z3) strongly elevated the RSL from 2 to 10 days (on average by 35.77%) when compared with Z2.

RV shown a high decrease from 2 to 4 days (on average by 14.51%) while a remarkable increase from 6 to 8 days (on average by 49.13%) in Q2 when relative to Q0; however, Q3 reversed the decreased RV caused by salinity from 2 to 8 days, though it was non-significant; notably, Q3 significantly increased RV at 10 days after salinity treatment (by 18.38%), when in comparison with Q2. In contrast to Chaoyouqianhao, RV exhibited a decrease trend during the treatment time course (on average by 26.06%) in Z2 when compared to Z0, whereas Z3 strongly enhanced RV from 8 to 10 days (on average by 81.29%).

The results obtained from Q2 shown that salinity slightly enhanced the growth of RAD (on average by 7.08%) from 2 to 10 days while Q3 slightly increased it (on average by 2.71%) from 4 to 8 days. Additionally, Z2 exhibited an average 6.1% decrease in RAD when compared to Z0, while Z3 restored it (on average by 9.72%) from 8 to 10 days.

### Effect of exogenous ABA on salt stress with respect to seedling biomass

**Figure 2:**
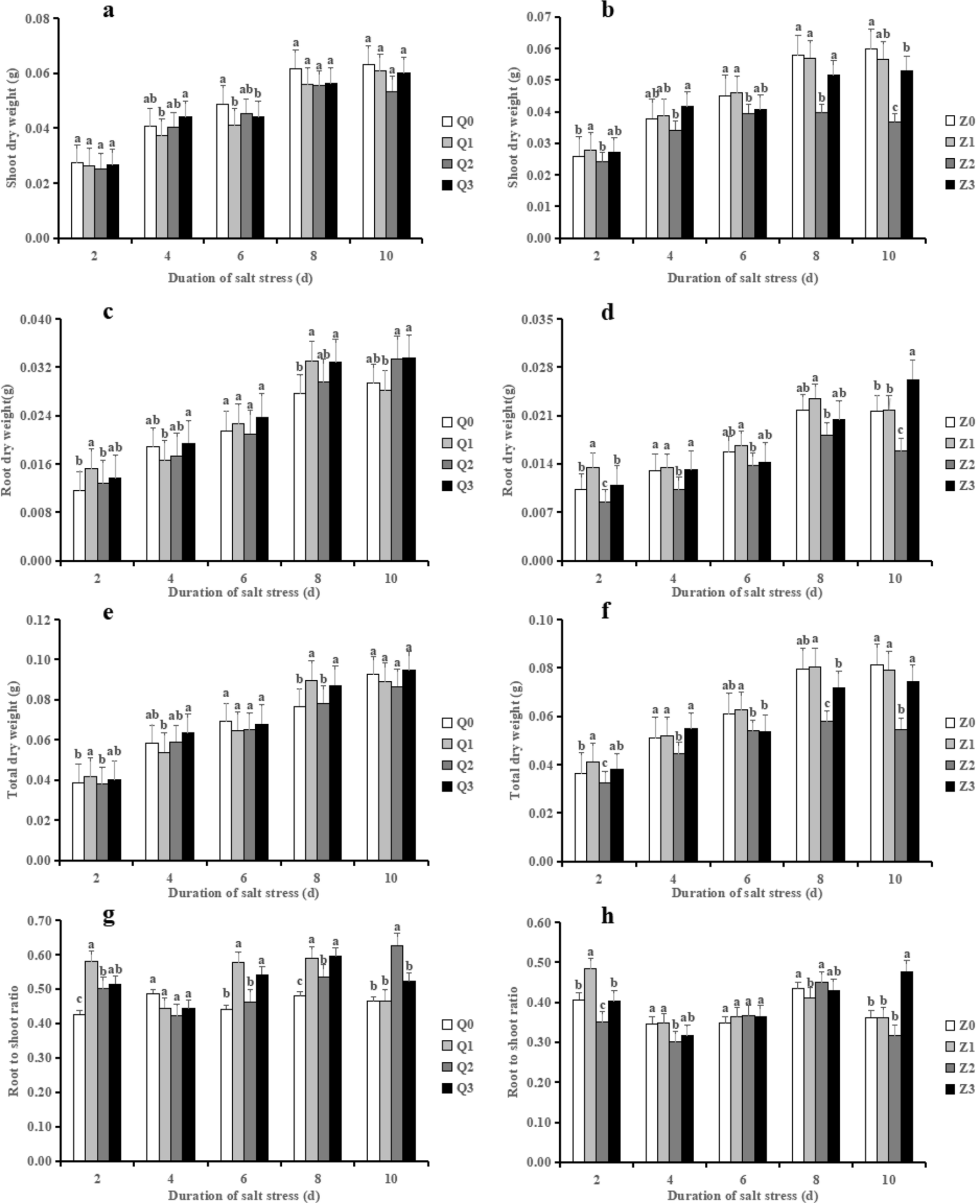
Effect of ABA with leaf sprayed on shoot dry weight (**a, b**), root dry weight (**c, d**), total dry weight (**e, f**) and root to shoot ratio (**g, h**) of two rice cultivar seedlings under salt stress. Values are shown as the mean ± standard and derived from four replicates (n = 4). Bars indicate standard errors. Different letters above the bars indicate significant differences at the five percent significant level according to Duncan's multiple range tests.

Salinity treatment exhibited a different trend in shoot dry weight, root dry weight, total dry weight and root to shoot ratio between Chaoyouqianhao and Huanghuazhan (Fig. 2a–h). When in comparison with the control, salinity treatment (Q2) decreased the shoot dry weight whereas Z2 showed a stronger decrease from 6 to 10 days, which were slightly reversed by Q3 and obviously reversed by Z3 (Fig. 2a, b), respectively. Q2 displayed a downward to an upward trend in root dry weight from 4 to 10 days when compared with Q0, whereas Q3 slightly increased root dry weight (on average by 8.77%) during the treatment time course (Fig. 2c). On the contrary, salinity treatment (Z2) strongly inhibited root dry weight (on average by 19.09%) during the treatment time course when compared to Z0, whereas Z3 prominently elevated it from 2 to 4 days (28.50%) and 10 days (65.12%) after salt stress, respectively (Fig. 2d). No significance was found in total dry weight among Q0, Q2 and Q3 (except at 8 days). Nevertheless, Z2 resulted in a significant decrease in total dry weight (on average by 18.86%) while Z3 strongly increased it (on average by 25.10%) in 2–10 days, in comparison with Z0 and Z2, respectively (Fig. 2f).

As shown in Fig. 2g, when in comparison with Q0, Q2 exhibited a slight increment in root to shoot ratio (on average by 8.18%), which was increased by ABA + NaCl treatment (Q3) (on average by 13.95%) during 6–8 days. With regard to Huanghuazhan, Z2 significantly reduced root to shoot radio (13.44% on average) from 2 to 4 days and a 12.78% decrease at 10 days, which was restored by ABA in Z3 (on average by 9.94%) from 2 to 4 days; notably, Z3 significantly enhanced root to shoot radio (on average by 51.00%) at 10 days when compared to Z2.

### Effects of exogenous ABA on leaf pigment contents in rice seedlings under salt stress

**Figure 3:**
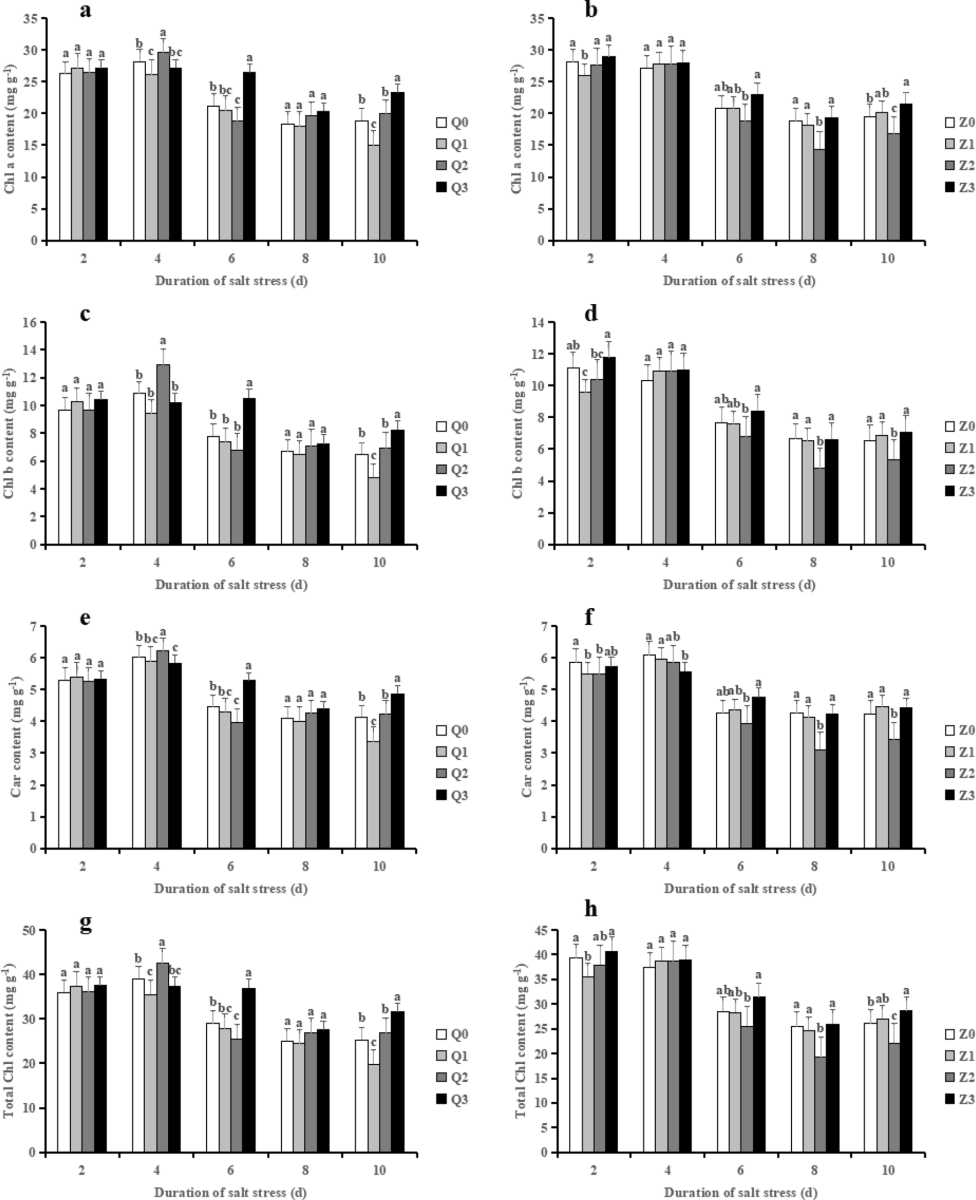
Effect of ABA with leaf sprayed on the chlorophyll a (**a, b**), chlorophyll b (**c, d**), carotenoid (**e, f**) and total chlorophyll contents (**g, h**) of two rice cultivar seedlings under salt stress. Values are shown as the mean ± standard and derived from four replicates (n = 4). Bars indicate standard errors. Different letters above the bars indicate significant differences at the five percent significant level according to Duncan's multiple range tests.

The chlorophyll contents displayed a different trend during the treatment time course in the two rice varieties (Fig. 3a–h). In comparison with Q0, Q2 significantly decreased chl a (11.34%), chl b (12.71%), Car (10.86%) and total chlorophyll content (11.71%); however, Q3 reversed the decreased chlorophyll contents caused by NaCl at 4 days, which were increased by 40.76%, 54.97%, 33.14% and 44.54%, respectively. A slight increased was observed in terms of chlorophyll contents in Q2 during 8–10 days whereas Q3 further enhanced them at 10 days. In contrast, Z2 strongly decreased the chlorophyll contents (on average by 15.84, 19.48%, 17.73% and 16.78%, respectively) when compared to Z0 during 6–10 days, which were significantly restored by Z3 (on average by 28.53%, 31.69%, 28.77% and 29.32%, respectively).

### Effects of exogenous ABA on photosynthetic parameters in rice seedlings under salt stress

**Figure 4:**
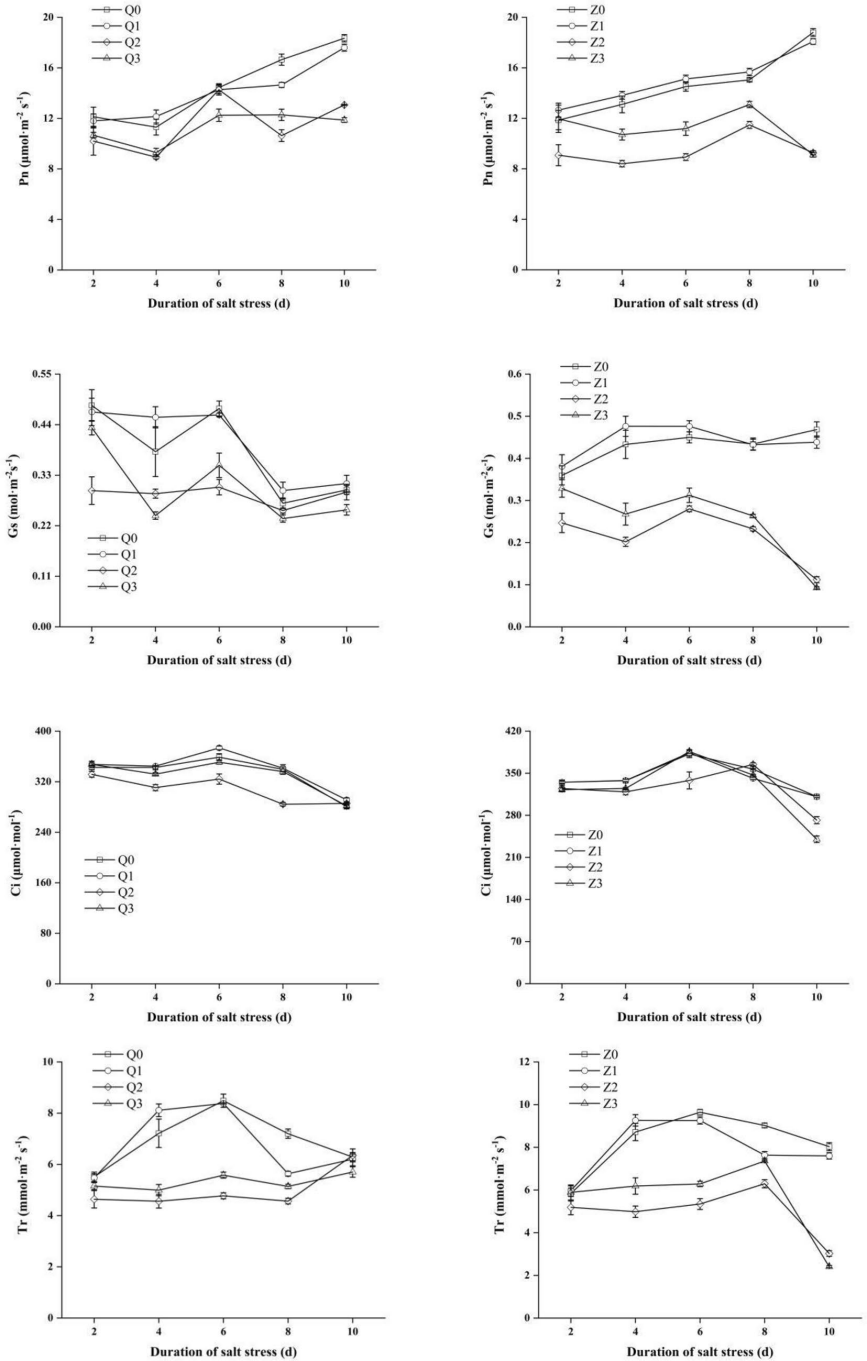
Effect of ABA with leaf sprayed on net photosynthetic rate (Pn), stomatal conductance (Gs), intercellular CO_2_ concentration (Ci) and transpiration rate (Tr) in leaves of two rice cultivar seedlings under salt stress. Values are shown as the mean ± standard and derived from four replicates (n = 4). Bars indicate standard errors. Different letters above the bars indicate significant differences at the five percent significant level according to Duncan's multiple range tests.

The *Pn*, *Gs*, *Ci* and *Tr* decreased in the two rice varieties in salinity treatments (Q2 or Z2) when compared with non-saline treatments (Q0 or Z0), with an average of 20.58%, 21.20%, 7.32% and 26.39%, respectively in Chaoyouqianhao and 34.44%, 48.99%, 6.11% and 38.22% respectively in Huanghuazhan during the treatment time course (Fig. 4). However, ABA + NaCl treatment (Q3) showed a strong improvement in *Pn* (15.44%) in the presence of saline during 8 days of salt stress compared with Q2, while Z3 showed a stronger improvement (on average by 24.62%) in 2–8 days when compared with Z2.

Similar to Pn, ABA showed an improvement in Gs with imposition of saline in comparison with Q2 at 2 days (46.48%) and 6 days (15.69%) of salinity treatment, respectively; while Z3 improved the Gs in 2–8 days (on average by 22.59%) when compared with Z2.

There were minor differences in Ci in the two varieties in NaCl + ABA treatments (Q3 or Z3) when compared with salinity treatments (Q2 or Z2) during treatment time course, except at 8 days of treatment in Q3 (an increase of 18.18%) and 6 days of treatment in Z3 (an increase of 14.20%) when compared with their respective control.

Moreover, ABA exhibited a different degree of enhancement in Tr in the presence of saline condition during 2–8 days, and the peak value of Tr was recorded at 6 days, which increased by 16.91% of control (Q2). Correspondingly, NaCl + ABA treatment (Z3) reversed the decreased Tr (on average by 18.05%) caused by saline during 2–8 days, when in comparison with saline alone treatment (Z2).

### Effects of exogenous ABA on fluorescence parameters in rice seedlings under salt stress

To assess the performance of PS II, chlorophyll fluorescence technique was used to determine some fluorescence parameters. Results showed that no significant difference between salinity treatment and the control group in Fv/Fm, Fv/Fo and Fm/Fo in both of two rice cultivars during the treatment time course (Table 3). Interestingly, Fv/Fm, Fv/Fo and Fm/Fo declined due to salinity treatment when compared with the control; conversely, NaCl + ABA treatment (Q3 or Z3) significantly improved Fv/Fm, Fv/Fo and Fm/Fo in 8 days after salinity.

**Table 3:** Effects of exogenous ABA on fluorescence parameters of five treatment time course in the presence of salinity.

Fluorescence parameters	Treatments	Duration of salt stress (days)				
		2	4	6	8	10
Fv/Fm	Q_0_	0.803 ± 0.002b	0.799 ± 0.005a	0.795 ± 0.001ab	0.787 ± 0.001ab	0.786 ± 0.002b
	Q_1_	0.810 ± 0.002ab	0.801 ± 0.003a	0.784 ± 0.007b	0.786 ± 0.003b	0.780 ± 0.003c
	Q_2_	0.809 ± 0.001ab	0.801 ± 0.004a	0.801 ± 0.004a	0.779 ± 0..006b	0.792 ± 0.001ab
	Q_3_	0.813 ± 0.004a	0.793 ± 0.005a	0.798 ± 0.002a	0.793 ± 0.003a	0.793 ± 0.002a
	Z_0_	0.796 ± 0.013a	0.798 ± 0.002ab	0.791 ± 0.003ab	0.783 ± 0.003a	0.777 ± 0.003b
	Z_1_	0.802 ± 0.002a	0.794 ± 0.002b	0.787 ± 0.002b	0.789 ± 0.002a	0.776 ± 0.003b
	Z_2_	0.804 ± 0.003a	0.797 ± 0.002ab	0.793 ± 0.002ab	0.742 ± 0.013b	0.792 ± 0.003a
	Z_3_	0.808 ± 0.001a	0.808 ± 0.007a	0.801 ± 0.006a	0.783 ± 0.006a	0.784 ± 0.004ab
Fv/F0	Q_0_	4.080 ± 0.043b	4.024 ± 0.143a	3.866 ± 0.030ab	3.688 ± 0.025ab	3.681 ± 0.044b
	Q_1_	4.270 ± 0.046ab	4.036 ± 0.094a	3.776 ± 0.039b	3.679 ± 0.057ab	3.554 ± 0.057c
	Q_2_	4.245 ± 0.024ab	4.058 ± 0.120a	4.060 ± 0.109a	3.625 ± 0.088b	3.803 ± 0.025ab
	Q_3_	4.393 ± 0.149a	3.862 ± 0.098a	3.962 ± 0.057ab	3.833 ± 0.060a	3.837 ± 0.038a
	Z_0_	4.076 ± 0.169a	3.962 ± 0.049ab	3.758 ± 0.029b	3.562 ± 0.065a	3.504 ± 0.065bc
	Z_1_	4.065 ± 0.048a	3.868 ± 0.045b	3.700 ± 0.050b	3.752 ± 0.052a	3.467 ± 0.061c
	Z_2_	4.104 ± 0.071a	3.928 ± 0.048ab	3.839 ± 0.044ab	3.020 ± 0.170b	3.825 ± 0.075a
	Z_3_	4.223 ± 0.039a	4.051 ± 0.043a	3.930 ± 0.075a	3.770 ± 0.044a	3.677 ± 0.064ab
Fm/F0	Q_0_	5.080 ± 0.043b	5.024 ± 0.143a	4.866 ± 0.030ab	4.688 ± 0.026b	4.681 ± 0.044b
	Q_1_	5.270 ± 0.046ab	5.036 ± 0.094a	4.669 ± 0.113b	4.679 ± 0.057b	4.554 ± 0.057c
	Q_2_	5.245 ± 0.024ab	5.058 ± 0.120a	5.060 ± 0.109a	4.625 ± 0.087b	4.803 ± 0.025ab
	Q_3_	5.393 ± 0.149a	4.862 ± 0.098a	4.962 ± 0.057a	4.897 ± 0.038a	4.837 ± 0.038a
	Z_0_	5.138 ± 0.086a	4.962 ± 0.049ab	4.758 ± 0.029a	4.608 ± 0.063a	4.504 ± 0.065bc
	Z_1_	5.065 ± 0.048a	4.868 ± 0.045b	4.700 ± 0.050a	4.752 ± 0.052a	4.467 ± 0.061c
	Z_2_	5.104 ± 0.071a	4.928 ± 0.048ab	4.750 ± 0.139a	4.041 ± 0.180b	4.827 ± 0.074a
	Z_3_	5.223 ± 0.039a	5.051 ± 0.043a	4.930 ± 0.075a	4.645 ± 0.115a	4.676 ± 0.064ab
NPQ	Q_0_	0.357 ± 0.050a	0.445 ± 0.035a	0.450 ± 0.042a	0.808 ± 0.055a	0.683 ± 0.088a
	Q_1_	0.292 ± 0.037a	0.368 ± 0.029a	0.453 ± 0.073a	0.608 ± 0.027b	0.716 ± 0.057a
	Q_2_	0.344 ± 0.027a	0.363 ± 0.015a	0.673 ± 0.076a	0.612 ± 0.037b	0.898 ± 0.092a
	Q_3_	0.362 ± 0.014a	0.447 ± 0.052a	0.604 ± 0.076a	0.500 ± 0.095b	0.862 ± 0.062a
	Z_0_	0.465 ± 0.022a	0.280 ± 0.036b	0.270 ± 0.020c	0.980 ± 0.027a	0.932 ± 0.104a
	Z_1_	0.404 ± 0.037ab	0.430 ± 0.025ab	0.326 ± 0.057bc	0.858 ± 0.052a	0.944 ± 0.129a
	Z_2_	0.327 ± 0.038b	0.663 ± 0.087a	0.542 ± 0.043a	0.668 ± 0.064b	0.940 ± 0.036a
	Z_3_	0.407 ± 0.043ab	0.460 ± 0.079ab	0.468 ± 0.042ab	0.577 ± 0.066b	0.733 ± 0.067a
Qp	Q_0_	0.318 ± 0.029a	0.326 ± 0.019a	0.280 ± 0.015c	0.312 ± 0.024ab	0.326 ± 0.024b
	Q_1_	0.246 ± 0.027a	0.240 ± 0.016b	0.300 ± 0.017bc	0.254 ± 0.021ab	0.338 ± 0.012ab
	Q_2_	0.246 ± 0.022a	0.282 ± 0.030ab	0.380 ± 0.036ab	0.243 ± 0.026b	0.405 ± 0.035a
	Q_3_	0.300 ± 0.029a	0.260 ± 0.026ab	0.413 ± 0.033a	0.345 ± 0.047a	0.384 ± 0.020ab
	Z_0_	0.376 ± 0.026a	0.368 ± 0.038bc	0.270 ± 0.029ab	0.414 ± 0.015a	0.362 ± 0.019a
	Z_1_	0.332 ± 0.018a	0.338 ± 0.026c	0.212 ± 0.054b	0.414 ± 0.015a	0.396 ± 0.021a
	Z_2_	0.230 ± 0.038b	0.526 ± 0.012a	0.378 ± 0.027a	0.367 ± 0.034ab	0.366 ± 0.019a
	Z_3_	0.422 ± 0.041a	0.447 ± 0.023ab	0.380 ± 0.019a	0.310 ± 0.025b	0.352 ± 0.012a
Qy	Q_0_	0.824 ± 0.002ab	0.820 ± 0.003ab	0.812 ± 0.002bc	0.812 ± 0.002a	0.806 ± 0.002a
	Q_1_	0.828 ± 0.002a	0.824 ± 0.002a	0.806 ± 0.002c	0.812 ± 0.002a	0.782 ± 0.028a
	Q_2_	0.818 ± 0.002b	0.812 ± 0.004b	0.820 ± 0.003ab	0.794 ± 0.013a	0.782 ± 0.033a
	Q_3_	0.830 ± 0.003a	0.818 ± 0.002ab	0.822 ± 0.004a	0.808 ± 0.007a	0.812 ± 0.004a
	Z_0_	0.822 ± 0.004a	0.818 ± 0.004a	0.814 ± 0.002a	0.808 ± 0.002a	0.794 ± 0.011a
	Z_1_	0.822 ± 0.002a	0.816 ± 0.002a	0.812 ± 0.002a	0.790 ± 0.023a	0.778 ± 0.025a
	Z_2_	0.828 ± 0.002a	0.810 ± 0.006a	0.794 ± 0.007b	0.764 ± 0.030a	0.656 ± 0.144a
	Z_3_	0.824 ± 0.002a	0.814 ± 0.002a	0.820 ± 0.003a	0.788 ± 0.015a	0.812 ± 0.004a
Rfd	Q_0_	0.533 ± 0.029a	0.654 ± 0.058a	0.643 ± 0.058b	1.060 ± 0.081a	0.948 ± 0.139a
	Q_1_	0.400 ± 0.055a	0.468 ± 0.041b	0.813 ± 0.041ab	0.786 ± 0.038bc	1.022 ± 0.080a
	Q_2_	0.468 ± 0.030a	0.537 ± 0.043ab	1.158 ± 0.094a	0.743 ± 0.048c	1.330 ± 0.166a
	Q_3_	0.478 ± 0.044a	0.607 ± 0.068ab	0.942 ± 0.139ab	0.957 ± 0.088ab	1.308 ± 0.114a
	Z_0_	0.674 ± 0.059a	0.480 ± 0.093c	0.442 ± 0.043b	1.378 ± 0.066a	1.266 ± 0.152ab
	Z_1_	0.606 ± 0.055a	0.700 ± 0.055c	0.464 ± 0.044b	1.308 ± 0.077a	1.407 ± 0.173a
	Z_2_	0.416 ± 0.082b	1.434 ± 0.098a	0.855 ± 0.054a	0.923 ± 0.072b	1.356 ± 0.062a
	Z_3_	0.686 ± 0.040a	1.07 ± 0.142b	0.863 ± 0.140a	0.864 ± 0.130b	0.970 ± 0.078b

Fv/Fm indicates maximal photochemical efficiency of PS II, Fv/Fo indicates potential activity of PS II, Fm/Fo indicates electron transfer case of PS II, NPQ indicates non-photochemical quenching coefficient, Qp indicates the coefficient of photochemical fluorescence quenching, Qy indicates effective photochemical efficiency of PS II, Rfd indicates fluorescence decrease ratio. Q_0_ treatment of water, Q_1_ treatment with ABA, Q_2_ treatment with NaCl, Q_3_ treatment with ABA + NaCl in Chaoyouqianhao. Z_0_ treatment of water, Z_1_ treatment with ABA, Z_2_ treatment with NaCl, Z_3_ treatment with ABA + NaCl in Huanghuazhan. Datas are mean ± standard error of at least four replicates. Within each column, different letters indicate significant difference at the five percent significant level according to Duncan's multiple range tests.

As showed in Table 3, Q2 decreased the Qp when compared with Q0 during 2–4 days and 8 days of salinity treatment; however, Q3 showed a remarkable increase (41.98%) in 8 days. No significant difference was observed among all the treatments in Huanghuazhan during the treatmrent time course except 2 days after salt stress. Similarly, no significant difference was found among all the treatments in Chaoyouqianhao during 2–10 days, though Q2 strongly increased NPQ when compared with Q0 and it was reversed by Q3 when compared with Q2, in 6 days and 10 days after salt stress. Likewise, when in comparison with Z0, Z2 showed a significant increase (on average by 118.77%) in NPQ during 4–6 days. In contrast, Z3 restored the increased NPQ caused by NaCl (on average by 22.14%). In comparison with Q0, salinity treatment (Q2) resulted in a slight reduction in Qy, while Q3 improved Qy during the treatment time course except 6 days (Table 3). A decline in terms of Qy was observed in Z2 (on average by 6.57%) when compared with Z0 whereas Z3 improved it (on average by 7.67%) during 4–10 days.

Rfd is able to characterize the leaf potential photosynthesis quantum transformation efficiency. Results showed that salinity treatment (Q2) reduced Rfd when compared with Q0, whereas Q3 reversed the reduction caused by NaCl in 2–4 days and 8 days. Notably, salinity treatment (Z2) displayed an upward to a downward trend in Rfd from 2 to 8 days in comparison with their respective control; however, Z3 exhibited a minor difference in Rfd during 6–8 days.

### Effects of exogenous ABA on ion homeostasis in rice seedlings under salt stress

**Figure 5:**
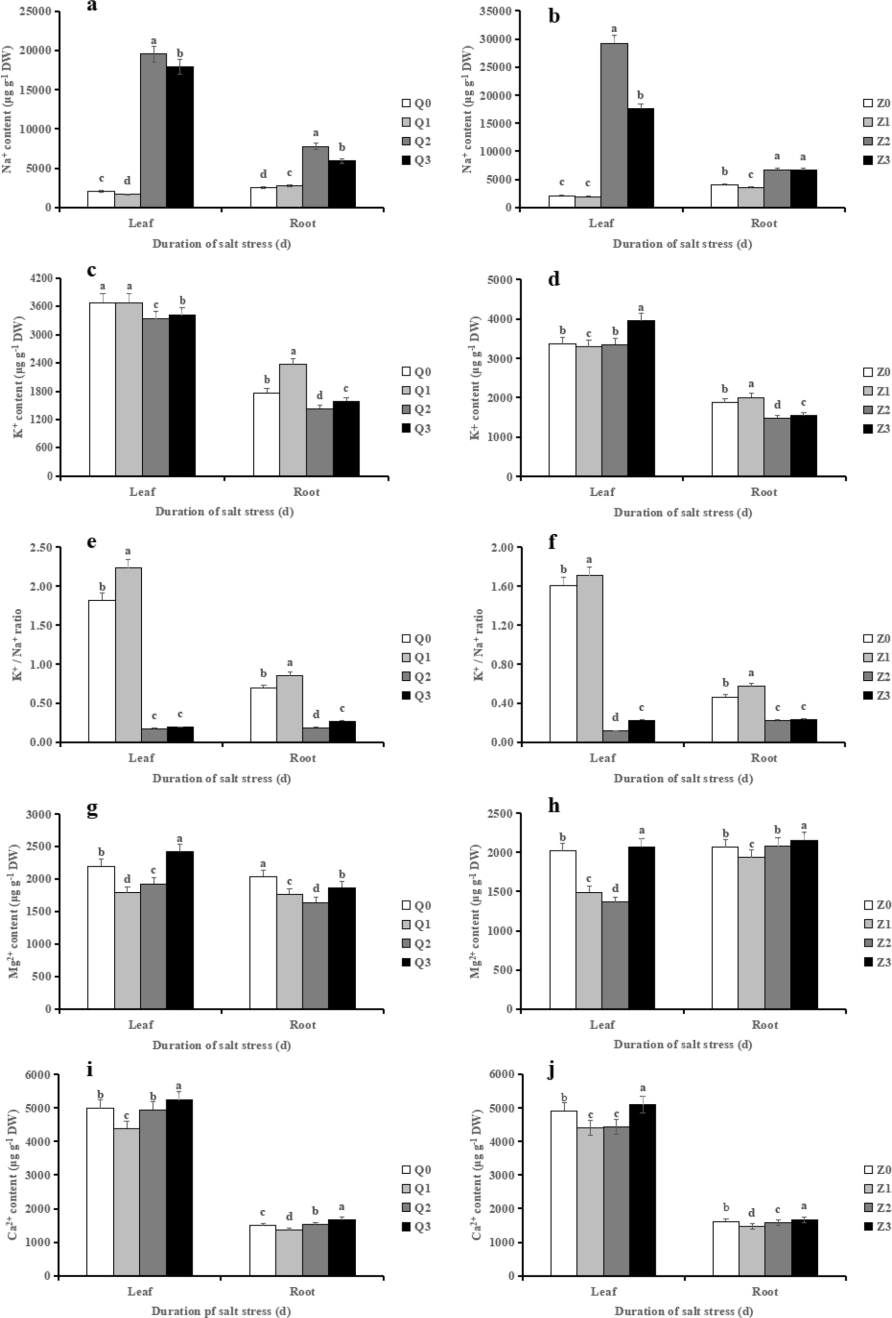
Effect of ABA with leaf sprayed on the contents of Na^+^ (**a, b**), K^+^ (**c, d**), K^+^/Na^+^ ratio (**e, f**), Mg^2+^ (**g, h**) and Ca^2+^ (**i, j**) in leaves and roots of two rice cultivar seedlings at 8 days after salt stress. Values are shown as the mean ± standard and derived from four replicates (n = 4). Bars indicate standard errors. Different letters above the bars indicate significant differences at the five percent significant level according to Duncan's multiple range tests.

Salt stress significantly increased the contents of Na^+^ in leaves and roots of two rice cultivars, which increased by 863.82 and 207.65%, respectively in Chaoyouqianhao and 1299.36 and 67.72%, respectively in Huanghuazhan (Fig. 5a, b). Compared with Q2, ABA + NaCl (Q3) significantly reduced the Na^+^ content (by 8.31% in leaves and 23.54% in roots, respectively) and Z3 significantly reduced it by 39.85% in leaves, whereas minor difference was found in roots when compared with Z2.

The contents of K^+^ in leaves and roots showed a significant decrease in salt-stressed rice seedlings when compared with non-stressed rice seedlings in terms of Chaoyouqianhao. However, Q3 significantly restored the decreased K^+^ content caused by salt stress (Fig. 5c, d). Similarly, Z3 significantly increased it in leaves (by 18.03%) and roots (by 4.05%) of Huanghuazhan. The K^+^/Na^+^ ratio in leaves and roots of the two rice cultivars exhibited a strongly decrease in presence of saline condition, due to the increase of Na^+^ and the decrease of K^+^ content, while ABA + NaCl treatment increased K^+^/Na^+^ ratio in leaves and roots under saline condition (Fig. 5e, f).

To further clarify the regulation of ABA on mineral homeostasis under salt stress, the contents of two important ions Mg^2+^ and Ca^2+^ were measured (Fig. 5g–j). Results showed that salinity treatment (Q2) significantly reduced Mg^2+^ content in leaves (by 12.42%) and roots (by 19.48%) in comparison to their respective control (Q0). The decrease of Mg^2+^ content was reversed by ABA + NaCl treatments (Q3), which increased by 25.43% in leaves and 13.82% in roots, respectively. Likewise, the decrease of Mg^2+^ content was observed in leaves but not in roots of Huanghuazhan under salt stress; nonetheless, Z3 exhibited a significant increase in the two tissue. Interestingly, the content of Ca^2+^ in roots significantly increased in Chaoyouqianhao under saline condition. Conversely, Z2 significantly reduced the Ca^2+^ content in roots when compared with Z0. Nevertheless, Q3 or Z3 showed an enhancement in the content of Ca^2+^ content, when compared with saline alone treatment (Q2 or Z2).

### Effects of exogenous ABA on salt stress in terms of membrane lipid peroxide (malondialdehyde) and H_2_O_2_ in rice seedlings

**Figure 6:**
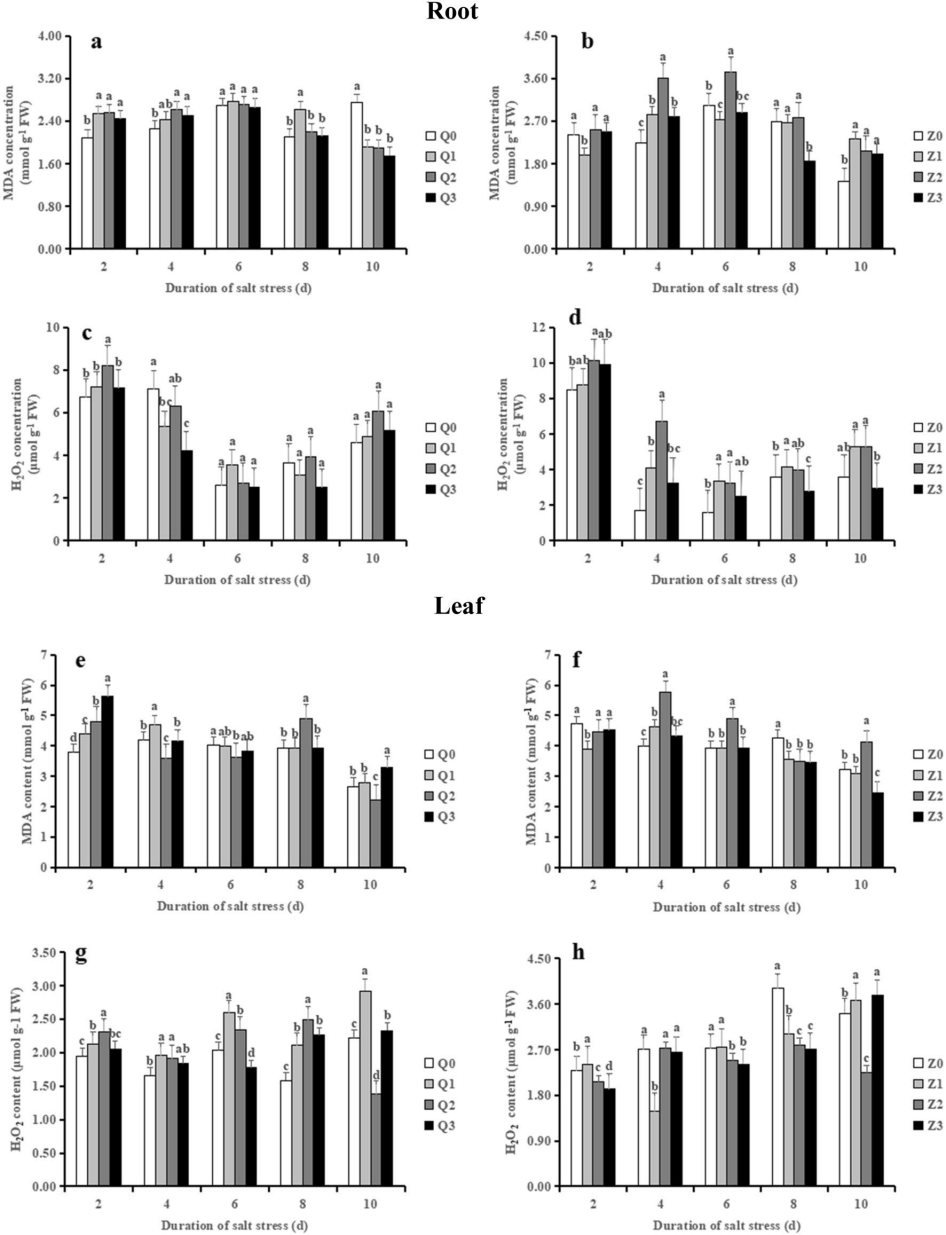
Effect of ABA with leaf sprayed on malondialdehyde and H_2_O_2_ concentration in roots and leaves of two rice cultivar seedlings under salt stress. Values are shown as the mean ± standard and derived from four replicates (n = 4). Bars indicate standard errors. Different letters above the bars indicate significant differences at the five percent significant level according to Duncan's multiple range tests.

The MDA contents in roots exhibited an upward to a downward trend in salinity treatment in both of two rice cultivars (Fig. 6a, b). Compared to Q0, MDA contents showed a significant increment in Q2 in roots (on average by 18.78%) from 2 to 4 days and in leaves (19.58%) at 8 days, respectively; however, no significance was found between Q3 and Q2 in MDA content of roots during the treatment time course while Q3 significantly reduced it in leaves compared with Q2 at 8 days (Fig. 6e). By comparison, Z2 strongly increased MDA level in roots (on average by 27.26%) during the treatment time course in comparison with Z0, which significantly decreased by Z3 (on average by 26.33%) from 4 to 8 days. Notably, Z2 showed a significant increase in MDA of leaves during 4–10 days except 8 days after salt stress, whereas Z3 reversed the increased MDA content (Fig. 6f).

The concentration of H_2_O_2_ in roots in all treatments during 2–4 days were higher than that of 6–10 days in general (Fig. 6c, d). Salinity treatment (Q2) shown a prominent elevation in H_2_O_2_ concentration in roots in the initial 2 days when relative to Q0, which was reversed by ABA in Q3; however, no significance was found among Q0, Q2 and Q3 from 6 to 10 days (Fig. 6c). The concentration of H_2_O_2_ in leaves under salt stress significantly increased (on average by 26.63%) in Q2, which was reversed by NaCl + ABA (Q3) during 2–8 days (Fig. 6g). In comparison with Z0, H_2_O_2_ in roots showed a strong increase in 2–10 days after salt stress; however, Z3 completely reversed (on average by 36.93%) the increased H_2_O_2_ caused by salinity only from 4 to 10 days (Fig. 6d). It was of note that the concentration of H_2_O_2_ in leaves significantly decreased in presence of salinity (Z2), whereas Z3 further decreased it, during 2–10 days except at 4 days.

### Effects of exogenous ABA on the antioxidant enzyme activities in rice seedlings under salt stress

**Figure 7:**
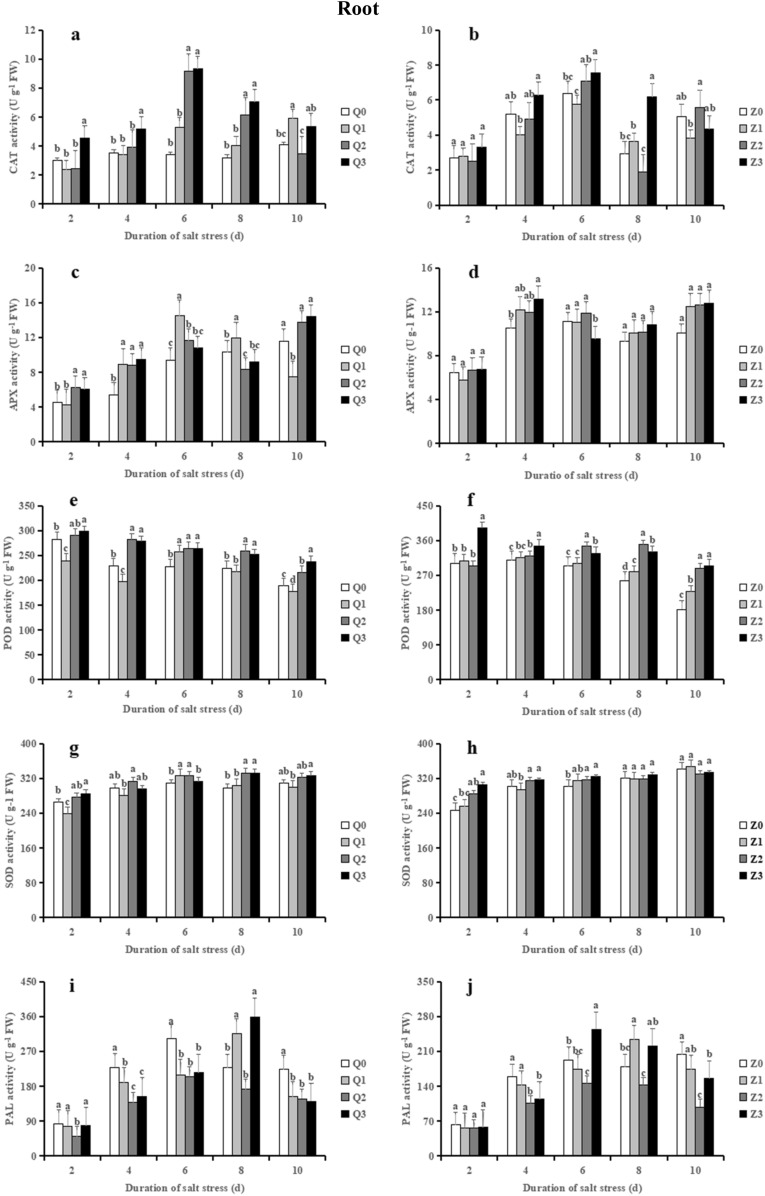

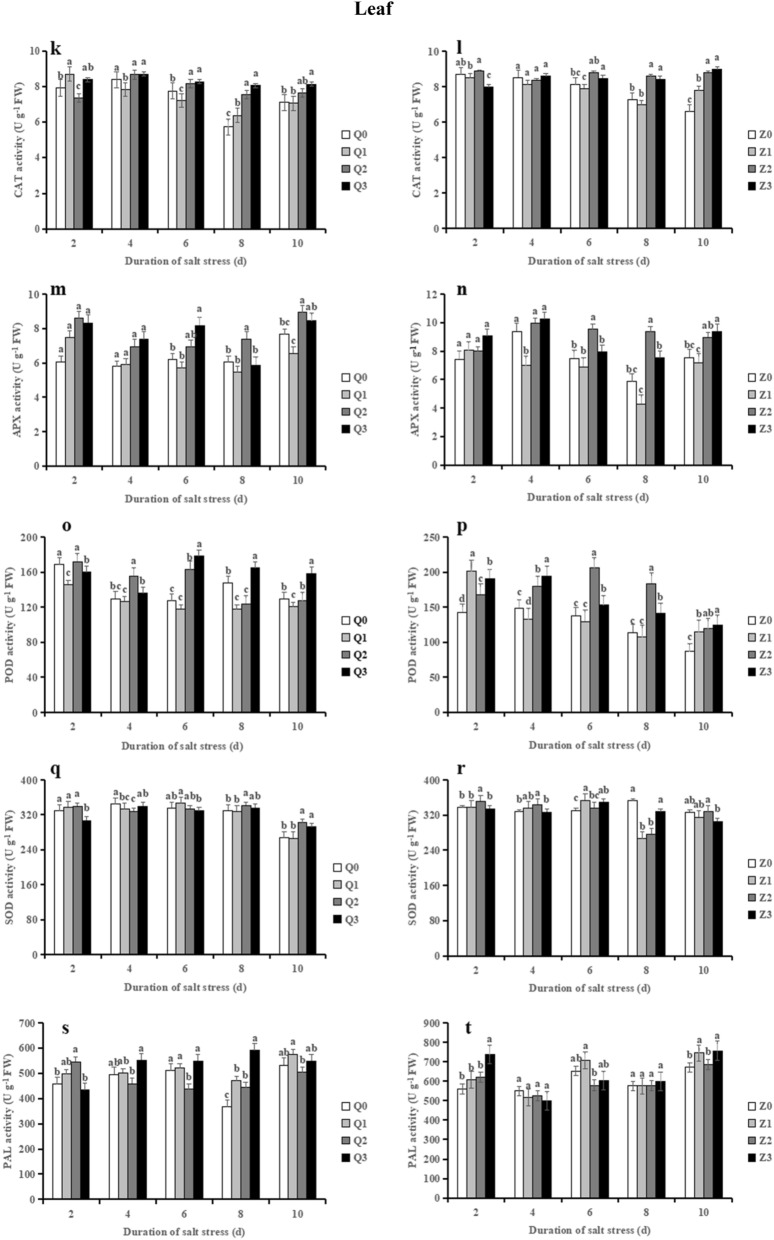
Effect of ABA with leaf sprayed on CAT, APX, POD, SOD and PAL in roots (**a–j**) and leaves (**k–t**) of two rice cultivar seedlings under salt stress. Values are shown as the mean ± standard and derived from four replicates (n = 4). Bars indicate standard errors. Different letters above the bars indicate significant differences at the five percent significant level according to Duncan's multiple range tests.

The CAT activities in roots showed increasing trends in the two rice cultivars from 2 to 6 days (Fig. 7a, b). When in comparison with the control (Q0), Q2 caused a strongly increase in CAT (on average by 90.50%) whereas Q3 further increased it by 16.01% on average, from 4 to 8 days. Notably, salinity treatment (Z2) showed a 34.60% decrease in CAT activities compared with Z0, whereas Z3 strongly reversed (223.78%) the decreased CAT caused by salinity at 8 days. Similarly, CAT activities showed a strong increase in Q2 (on average by 14.85%) and Z2 (on average by 20.11%) from 6 to 10 days in leaves whereas no significance was found in ABA + NaCl treatment (Q3 and Z3) (Figs. 7k and l).

In comparison with Q0, salt stress treatment (Q2) resulted in a remarkable increase in APX activity in roots (on average by 41.21%) while Q3 weakly reversed it during 2–6 days of salt stress (Fig. 7c). Besides, the APX activity in roots shown no significance among Z0, Z2 and Z3 during the treatment time course except at 6 days (Fig. 7d). In comparison with the controls, APX activity in leaves displayed a stronger increment than that in roots in salinity treatment alone during the treatment time course (Fig. 7k, l). Notably, Q3 further enhanced APX activity (on average by 10.55%) in 4–6 days whereas Z3 further enhanced it (on average by 8.17%) in 2–4 days in comparison with their respective control.

The average activities of POD in roots remained relatively stable between the two rice cultivars (Fig. 7e, f). When relative to the controls (Q0 and Z0), both salinity (Q2 and Z2) and ABA + NaCl treatments (Q3 and Z3) significantly elevated POD activities from 4 to 10 days. POD activities in leaves showed a significant increase in Q2 when relative to Q0 from 2 to 6 days, and ABA treatment further increased it (9.60%) at 6 days (Fig. 7o). Similarly, Z2 significantly enhanced the POD activity in leaves during the treatment time course when compared to Z0, whereas Z3 further enhanced it from 2 to 4 days (Fig. 7p).

Under saline condition (Q2), SOD activity in roots exhibited a gradual increase trend (on average by 6.17%) during the treatment time course in comparison with Q0; however, no significance was found between Q3 and Q2 in general (Fig. 7g). Similarly, Z2 showed an average 8.10% increase in SOD activity in roots when compared with Z0 during 2–6 days, whereas a slight average 1.75% decrease in it from 8 to 10 days. However, no significance was found between Z3 and Z2 (Fig. 7h). In comparison with the control, salt stress significantly decreased the SOD activity in leaves at 4 days, which was restored by ABA in the ABA + NaCl treatment (Fig. 7q). The SOD activity exhibited a strong decrease when compared with the normal condition (Z0) at 8 days whereas Z3 showed a significant increment in it in comparison with Z2 (Fig. 7r).

In comparison with the controls (Q0 or Z0), salinity treatment showed a strong decrease in PAL activity in roots (on average by 34.11 % in Q2 and 27.95% in Z2, respectively) from 2 to 10 days (Figs. 7i and j). Obviously, Q3 gradually increased the PAL levels (on average by 45.51%) in 2 to 8 days relative to Q2 and the maximum range was observed at 8 days, whereas Z3 significantly enhanced the PAL activity (on average by 49.09%) in 4 to 10 days compared with Z2, and the maximum range was recorded at 6 days. As showed in Fig. 7s, Q2 decreased the PAL activity in leaves in 4 to 6 days and 10 days when compared with Q0; notably, Q3 exhibited a significant increase in PAL from 4 to 10 days. No significance was found in PAL activity between Z0 and Z2, whereas Z3 prominently increased it at 2 days and 10 days, when compared with Z2 (Fig. 7t).

### Effects of exogenous ABA on the activities of AsA and GSH contents in rice seedlings under salt stress

**Figure 8:**
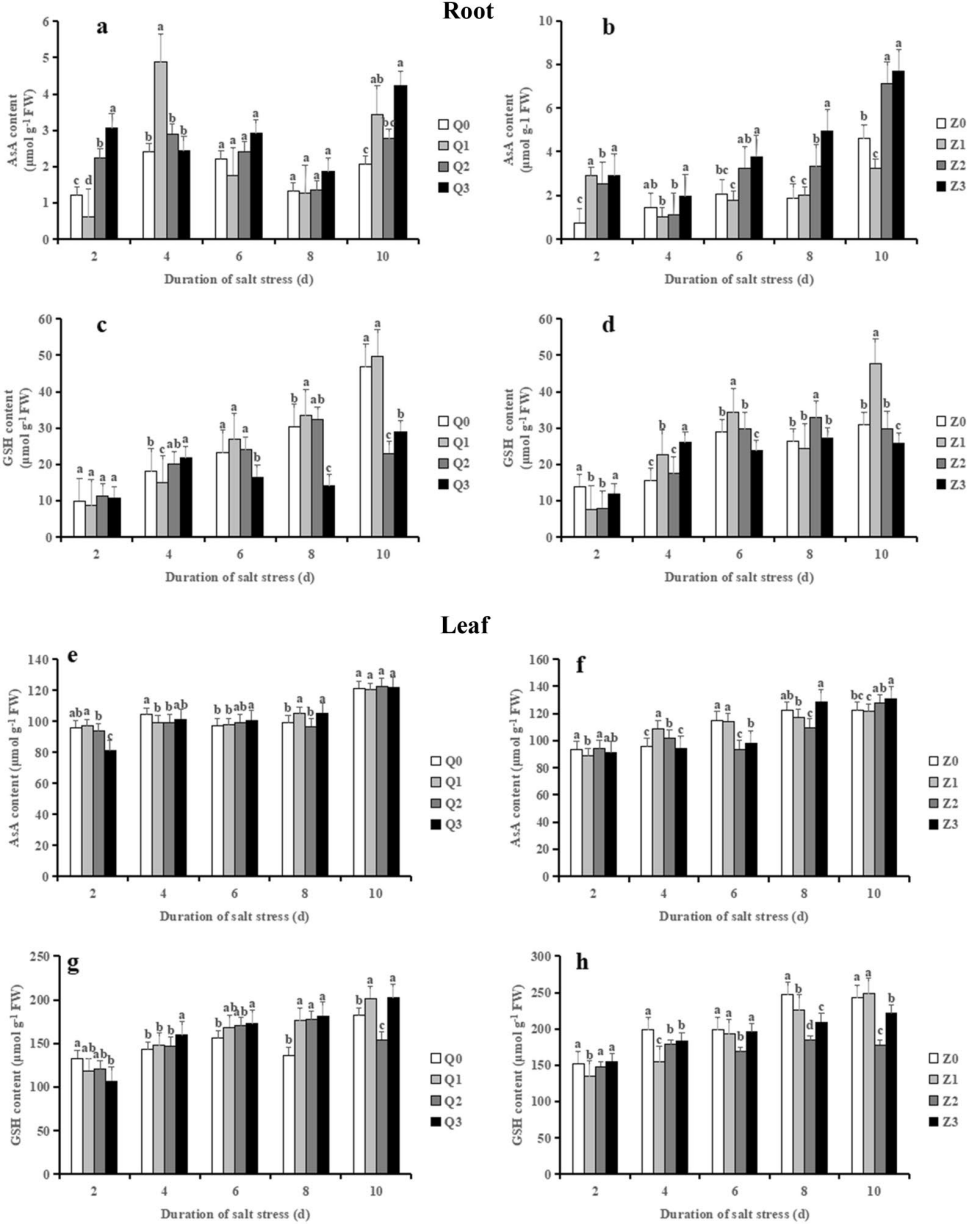
Effect of ABA with leaf sprayed on AsA and GSH contents in roots (**a–d**) and leaves (**e–h**) of two rice cultivar seedlings under salt stress. Values are shown as the mean ± standard and derived from four replicates (n = 4). Bars indicate standard errors. Different letters above the bars indicate significant differences at the five percent significant level according to Duncan's multiple range tests.

The levels of AsA in roots displayed a significant increment in Q2 (by 85.89%) and Z2 (by 237.39%) compared with Q0 and Z0, respectively; which was further enhanced by ABA in Q3 (by 37.16%) and Z3 (by 14.30%), respectively in the initial 2 days of salt stress (Fig. 8a, b). Notably, the AsA contents shown increasing trends in Q2 from 6 to 10 days, which increased by an average 39.60% in comparison with Q0; moreover, ABA + NaCl treatment further increased AsA contents (on average by 24.12%) when compared with Q2 in Chaoyouqianhao. Conversely, salinity treatment (Q2 and Z2) decreased the AsA contents when compared with their respective controls, which decreased by 2.53% and 10.51%, respectively at 8 days (Figs. 8e and f); ABA + NaCl completely reversed the decreased AsA contents caused by NaCl (increased by 9.03% in Q3 and 17.45% in Z3).

The GSH contents in roots showed an increasing trend during the treatment time course (Fig. 8c, d). Compared with Q0, Q2 displayed minor differences in GSH levels (on average by 5.25%), whereas Q3 significantly reduced the levels of GSH (on average by 44.03%) in 6–8 days, relative to Q2. Likewise, an average 14.13% increase in GSH levels was observed in Z2, which was reversed by ABA in Z3 (on average by 18.5%) in 6–8 days, reached to a significant level. Under salinity alone treatment, GSH contents in leaves displayed an opposite trend between the two rice cultivars (Figs. 8g and h). When compared with Q0, Q2 significantly enhanced GSH contents (on average by 19.44%) in 6–8 days, whereas the levels of GSH showed minor differences between Q3 and Q2. However, Z2 significantly decreased GSH contents (on average by 20.21%) in 6–8 days, whereas Z3 showed a strong increase in GSH contents (on average by 14.97%) in relative to Z2.

### Effects of exogenous ABA on the levels of soluble protein in rice seedlings

**Figure 9:**
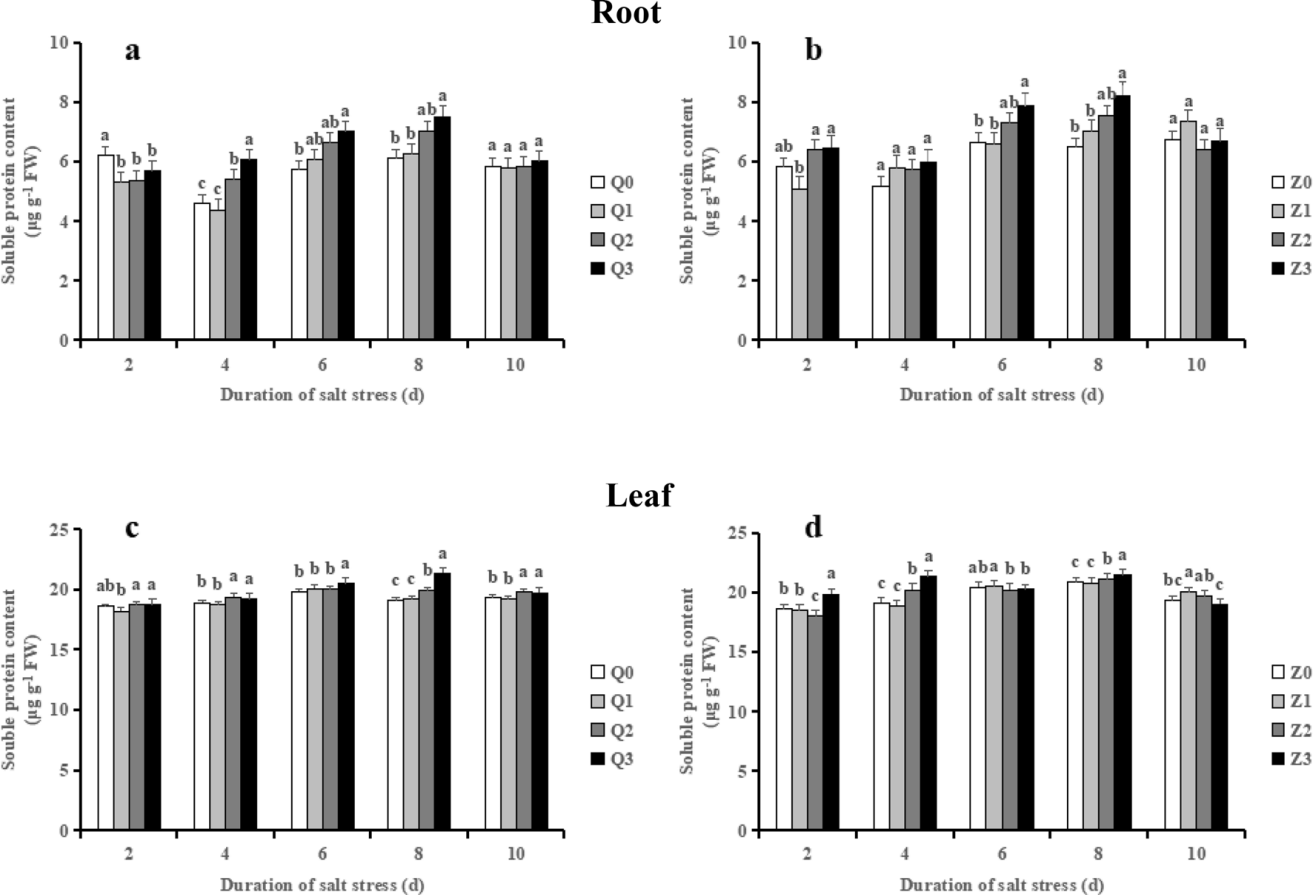
Effect of ABA with leaf sprayed on soluble protein content in roots (**a, b**) and leaves (**c, d**) of two rice cultivar seedlings under salt stress. Values are shown as the mean ± standard and derived from four replicates (n = 4). Bars indicate standard errors. Different letters above the bars indicate significant differences at the five percent significant level according to Duncan's multiple range tests.

The soluble protein contents in roots exhibited increasing trends from 4 to 8 days of the two rice cultivars (Fig. 9a, b). When compared with the controls (Q0 or Z0), salinity treatment exhibited an average 16.05% increase in Q2 and 12.16% in Z2, respectively, which were further enhanced by ABA in Q3 (on average by 8.41%) and Z3 (on average by 7.12%), respectively in 4–8 days.

Salinity alone treatment (Q2 or Z2) significantly increased the soluble protein contents in leaves in comparison with their controls, and Q3 and Z3 further enhanced the levels of soluble protein by 7.32% and 1.69%, respectively at 8 days (Figs. 9c and d).

### Effects of exogenous ABA on endogenous phytohormone in rice seedlings

Foliar spraying ABA alone treatment strongly increased the contents of ACC and IAA by 105.23% and 39.88%, respectively in Chaoyouqianhao and 27.09% and 170.18% respectively in Huanghuazhan when in comparison with their respective control (Table 4). Salinity treatment (Q2) showed an increase in ACC (72.33%), TZ (0.9%), IPA (93.12%), IAA (33.34%) and ABA (23.33%) level when compared with Q0. On the contrary, ABA further enhanced them by 212.00%, 13.21%, 13.75%, 10.89% and 57.46%, respectively in the presence of salinity (Q3). Interestingly, salinity treatment (Z2) showed a decrease in ACC (5.52%), TZ (17.42%), IPA (16.76%) and IAA (7.92%) while an increase in ABA (31.83%) contents when compared to their control. Nonetheless, ABA enhanced them by 317.80%, 80.73%, 122.02%, 21.69% and 80.98%, respectively in the presence of salinity (Z3) when compared to Z2.

**Table 4:** Effects of exogenous ABA on endogenous phytohormone at 8 days after salinity.

Treatments	ACC	TZ	IPA	IAA	ABA
	(nmol kg^−1^ Fresh Weight)	(nmol kg^−1^ Fresh Weight)	(nmol kg^−1^ Fresh Weight)	(nmol kg^−1^ Fresh Weight)	(nmol kg^−1^ Fresh Weight)
Q0	110.719 ± 8.932c	0.105 ± 0.012ab	2.878 ± 1.093ab	33.084 ± 2.632b	32.591 ± 1.265b
Q1	227.230 ± 8.914b	0.080 ± 0.005b	1.033 ± 0.239b	46.279 ± 5.149a	37.593 ± 1.685b
Q2	190.803 ± 5.144b	0.106 ± 0.007ab	5.558 ± 0.075a	44.115 ± 2.028a	40.114 ± 3.781b
Q3	593.054 ± 39.114a	0.120 ± 0.010a	6.322 ± 1.884a	48.919 ± 1.710a	63.162 ± 2.113a
Z0	240.064 ± 4.576b	0.132 ± 0.004b	6.057 ± 0.395b	34.604 ± 1.231a	31.456 ± 0.968c
Z1	305.088 ± 10.008b	0.075 ± 0.021c	4.024 ± 0.272b	93.494 ± 59.561a	36.436 ± 2.706bc
Z2	226.801 ± 6.317b	0.109 ± 0.003bc	5.042 ± 0.597b	31.863 ± 1.265a	41.468 ± 0.580b
Z3	947.567 ± 50.871a	0.197 ± 0.003a	11.194 ± 1.133a	38.774 ± 0.657a	75.048 ± 2.087a

ACC indicates 1-Aminocyclopropanecarboxylic acid, TZ indicates trans-Zeatin, IPA indicates N6-isopentenyladenosine, IAA indicated Indole-3-acetic acid, ABA indicates ( +)-Abscisic acid. Q_0_ treatment of water, Q_1_ treatment with ABA, Q_2_ treatment with NaCl, Q_3_ treatment with ABA + NaCl in Chaoyouqianhao. Z_0_ treatment of water, Z_1_ treatment with ABA, Z_2_ treatment with NaCl, Z_3_ treatment with ABA + NaCl in Huanghuazhan. Values are mean ± standard error of three replicates (n = 3). Within each column, different letters indicate significant difference at the five percent significant level according to Duncan's multiple range tests.

## Discussion

Plants were exposed to short-term salt stress triggers osmotic stress, resulting in reduced water absorption, redox imbalance, stomatal closure, and inhibiting new leaf growth and development of root systems^32^; however, long-term salinity causes ionic toxicity due to Na^+^ accumulation in adult leaves, caused premature senescence, thus reduced the rate of photosynthesis and nutrient accumulation^33^. Previous studies have shown that excessive salinity significantly inhibited plant growth and biomass accumulation, resulted in reduced plant growth and photosynthetic parameters^9,34^. Rachmawati et al. demonstrated that the reduced root surface area under salt stress lead to reduced water absorption, further inhibited cell division process and cell enlarged, resulting in reduced root length and root biomass^35^.

In the present study, salinity treatment (50 mM NaCl) significantly reduced shoot and root growth morphological indexes of the two rice varieties. Notably, during 8–10 days, salinity treatment increased the root biomass of Chaoyouqianhao, while the root biomass strongly decreased in Huanghuazhan (Figs. 2c and d), which also similar to previous result^36^. However, application of ABA partially or completely reversed the decrease of these indexes, i.e., the plant height, stem diameter, leaf area, root growth (RL, RSA, RV, RAD) and biomass of shoots and roots (Table 1). Similar results was observed in trifoliate orange^9^ and wheat^15^. Plant chlorophyll content is widely used as an indicator of the level of abiotic tolerance. Ample evidence showed that plants were exposed to stress environments like salinity resulted in reduced chlorophyll content, resulting in overall growth retardation^37^. In fact, the change of photosynthetic pigment, especially chlorophyll content under salt stress conditions still remained controversial and no clear conclusion has been drawn. It is demonstrated that the increase in chlorophyll content is temporary, mainly due to the positive effect of salinity^38^, At present, we found that salinity treatment exhibited an increase trend in chlorophyll a, chlorophyll b, carotenoid and total chlorophyll contents except at 6 days after salt stress, in Chaoyouqianhao (Fig. 3a, c, e, g), which consistent with the results of Gadelha et al., who demonstrated that the increased chlorophyll a, b and total chlorophyll contents were observed in rice seedlings under salt stress (80 mM NaCl) for 6 days^39^. Meanwhile, the decreased in these photosynthetic pigments in Huanghuazhan may be explained as the enhancement of chlorophyll-lase activity caused by salinity and enable chlorophyll breakdown^40^. It is speculated that the differences in chlorophyll content are related to the cultivar characteristics. On the other hand, ABA + NaCl significantly enhanced the photosynthetic pigments in 6–10 days in both of the two rice varieties (Figs. 3a–h). These results implied that ABA reduced the damage of salt stress to the photosynthetic cytochrome protein complex, and thus improved the photosynthetic capacity of rice.

Salt stress greatly inhibited biomass accumulation, resulting in reduced leaf area and photosynthetic parameters^34,41^. Hu et al demonstrated that photosynthetic parameters, Qy and Qp significantly decreased in the presence of salinity^9^. A similar result was observed in our study, showing that salt stress reduced Pn, Gs, Ci and Tr in the two rice genotypes (Fig. 4). Notably, the decreased photosynthetic parameters in Huanghuazhan was greater than that in Chaoyouqianhao, which was in harmony with that obtained by previous study^39^. Shibata et al. explained that the reduction in Fv/Fm caused by salinity was due to the photosynthetic electron transfer after light capture not in time, but rather directly consumed light energy via chlorophyll conversion, leading in a reduction in PSII^42^. In the present study, we found that salinity inhibited Fv/Fm, Fv/Fo, Fm/Fo and Qp at 8 days after salt stress, whereas NPQ significantly increased in rice seedlings especially Huanghuazhan (on average by 118.77%) in 4–6 days (Table 3), the present results were in agreement with that of previous study^43^. The stronger increase in NPQ of Huanghuazhan demonstrated that the absorption and conversion process of chlorophyll were improved to disperse the heat to a greater extent to avoid light damage^44^.

These results suggested that salt stress could reduce the biomass of rice shoots and roots via inhibiting photosynthesis and fluorescence processes as well as reducing the amount of photosynthetic pigments.

The salinity-induced damage to rice is mainly caused by Na^+^^33^. Excessive Na^+^ caused ion imbalance (also known as ion poisoning) in plants^45^. Results of Tavakkoli et al. showed that the absorption of nitrogen, phosphorus, calcium and other nutrients reduced under salt stress, and damaged the aboveground tissue function, especially by inhibiting the photosynthesis of chloroplasts^46^. In this study, we made a comprehensive assessment on morphological and physiological attributes in 2–10 days, and we found the optimal attributes were recorded at 8 days after salinity, thus we collected samples at the 8th day for ion analysis. In the present study, salinity stress triggered a large amount of Na^+^ contents while reduced K^+^ contents in roots and leaves of the two rice varieties (Fig. 5). Specially, the large influx of Na^+^, activating plasma membrane depolarization, in turn activates the K^+^ efflux channels, resulting in K^+^ efflux and increases Na^+^/K^+^ in the cytoplasm. Similar results were found in a research of melatonin application on rice under salt stress^47^. Notably, the accumulation of Na^+^ contents in leaves was far greater than that in roots, which may be attributed to that Na^+^ can only return to the root via the phloem, although Na^+^ is transported to the shoot via the xylem, suggesting that Na^+^ is strongly unidirectional, leading to the accumulation of Na^+^ in the leaves so that leaves are more susceptible to Na^+^ than the roots^48^. Moreover, Na^+^ contents in leaves of Huanghuazhan were more than that of Chaoyouqianhao, which was in harmony with those obtained by Chuamnakthong et al., who showed that the aboveground part of salt-tolerant rice has a strong Na^+^ efflux capacity as well as a low Na^+^/K^+^ when compared with salt-sensitive rice^49^. For example, the Na^+^ content accumulated in the leaves of salt-sensitive cv. IR29 was 5 ~ 10 times that of the salt-tolerant rice cultivars BK, FL478, or Pokkali. Similar results were also found in a result of previous research^39^. Both Ca^2+^ and Mg^2+^ are essential nutrients in the process of plant growth and development and participate in many important physiological regulation processes. For example, Ca^2+^ plays an important role in the stability of cell wall, cell membrane, membrane-binding protein, the regulation of inorganic ion transport and the second messenger in cell physiological and biochemical reactions^50^. Mg^2+^ is the main mineral element of chlorophyll, which directly affects the photosynthesis of crops, additionally, Mg^2+^ could participate in carbon fixation and carbon metabolism as coenzyme factor in plants. The present study showed that Mg^2+^ and Ca^2+^ contents were inhibited by salinity in leaves of two varieties, whereas Ca^2+^ contents significantly increased in the roots of Chaoyouqianhao. It has been shown that the cytosolic Ca^2+^ concentration reduced under salt stress conditions, while many studies have shown the enhanced levels of cytosolic Ca^2+^ under salt stress^51^. Results suggested that the cytosolic Ca^2+^ concentration was not uniform and it could vary with species and type of cells^51^.

Our results suggested that excluding Na^+^ ions, maintaining the low Na^+^ content in the roots and shoots is an important mechanism for plants tolerating high salinity.

Free radicals such as O_2_^−^, OH^−^ and H_2_O_2_ are known to be involved in regulating various signaling cascades associated with multiple biological functions of plants^52^. However, high concentration of free radicals-induced lipid peroxidation has been widely used in ROS to promote cell membrane damage in different stress environments and the MDA levels produced during membrane lipid peroxidation are often used as an indicator of oxidative damage^53^. Enzymatic-antioxidants and non-enzymatic antioxidants are an essential strategy against the environmental stresses. The former enzymatic-antioxidants including SOD, POD, CAT and APX, and the latter such as AsA and GSH directly involved in ROS homeostasis and detoxification in plant cells^5^. In the present study, salinity stress triggered an increase of MDA in roots and leaves of Chaoyouqianhao, while the greater increased MDA in roots and leaves of Huanghuazhan were observed towards the changed treatment time (Fig. 6); this results implied that salt stress causes a higher degree of peroxidative damage to the leaf and root tissues in Huanghuazhan than that in Chaoyouqianhao. Meanwhile, the contents of MDA in leaves were more than that in roots suggested that salinity stress caused a greater damage to leaves than to roots. H_2_O_2_ is widely involved in plant metabolism (e. g., cell wall biosynthesis) and plays central roles in many plant signaling pathways, including stress sensing, photosynthesis modulation, pathogen response, programmed cell death, hormonal action, and plant growth and development^54–56^. However, H_2_O_2_ can also be converted into harmful hydroxyl radicals by the fenton-type reaction, and then exerts multiple toxic effects in different cellular compartments^57^. Our study showed that salinity toxicity triggered increased H_2_O_2_ content in roots of Chaoyouqianhao and Huanghuazhan. Notably, the decreased H_2_O_2_ content due to salinity toxicity was observed in leaves of Huanghuazhan, similar results were obtained in barley^58^. Even so, Ye et al. showed that low H_2_O_2_ content was observed in salt-tolerant varieties^59^. Sadak declared that H_2_O_2_ played a key role in the improvement of salt stress by enhancing multiple defense-related mechanisms, with H_2_O_2_ molecules penetrating through the plasma membrane into different cellular compartments to mitigate the deleterious effects of salinity as a central role in activating various signal transduction pathways^60^. Furthermore, different studies have also demonstrated its involvement in triggering various genes, transcription factors, and phytohormones associated with salt stress tolerance^61,62^. All of these explained for the reason of the greater H_2_O_2_ in leaves of Chaoyouqianhao than that of Huanghuazhan in our study, as similarly reported by previous study^63^.

Previous studies have demonstrated that salinity stress activated the antioxidant systems and conferred salt tolerance^64–66^. Nevertheless, effects of salt stress on plant antioxidant systems are controversial. Qiu et al. showed that the activity of SOD, POD, CAT, APX decreased and the contents of GSH and AsA increased under salt stress in wheat seedlings^66^. Altaf et al. demonstrated that salt stress significantly increased SOD, CAT, APX activity and the level of GSH and AsA in tomato seedlings^65^. Our study showed that salinity increased the level of CAT, APX, POD and SOD in roots and leaves, particularly at 6 days or 8 days (Fig. 7). Interestingly, the increased CAT, APX and POD activity in leaves were more prominently than that in roots imposition with salinity. Similar results were observed in lentil seedlings^28^. This results demonstrated that the higher antioxidant enzyme activity in leaves could eliminate ROS and MDA so that reduced membrane damage when compared with root, which is also corresponded to the accumulation of more MDA and Na+ content in leaves. Besides, salinity increased stronger POD activity in roots, which similar to previous study of Meneguzzo et al., who demonstrated that APX activity was significantly higher in the root tissue than in the shoots of wheat seedlings^67^. Notably, the slight increased SOD in roots and leaves were observed at the early stage of salt stress, which in line with many previous studies^68,69^. This phenomenon may be due to that a non-enzymatic pathway was used to convert O_2_^−^ to H_2_O_2_ using antioxidants such as GSH and AsA. Last but not least, we found that lower H_2_O_2_ and MDA contents in leaves were maintain in Huanghuazhan at the late stage of salt stress (Figs. 6f and h), which was corresponded to the lower SOD activity (Fig. 7r), indicated that the higher activities of CAT, APX and POD in leaves participated in the decomposition of H_2_O_2_, thus reducing the H_2_O_2_ level and membrane damage.

The AsA-GSH cycle is considered as one of the most important nonenzymatic antioxidant systems^70^. The present study of enhancement of AsA and GSH contents in roots corroborated with the findings of previous research^71^. It was of note that AsA and GSH contents in leaves of the two rice genotypes inclined to reduce at the late stage of salt stress. As the most abundant antioxidant in plants, AsA is widely involved in the elimination of ROS during stress response so that protect the major macromolecules from oxidative damage^72^. Thus, this may explained that a greater oxidative damage to leaves than to roots. On the other hand, the decreased GSH may be attributed to the elimination of ROS in plant cells by depletion of GSH.

Plant hormones play a crucial role in plant growth and development. To mitigate salt stress at different developmental stages, the levels of plant hormones such as ABA, IAA, GA, JA, BR, SA and ethylene were modulated the physiological response of plants under salt stress^73^. In this study, we collected samples at the 8th day for endogenous hormones analysis. Hu et al demonstrated that salt stress significantly reduced IAA content and significantly increased zeatin riboside^9^, similar to our study, showing that ACC, IPA (a cytokinin of bound state), TZ (a natural plant cytokinin) and IAA reduced under salinity in Huanghuazhan. Lin and Kao demonstrated that there was no increase in ABA content in the roots under salinity stress, suggested that the inhibition of root growth in rice seedlings is unlikely to be mediated by ABA, however, this does not mean that the amount of ABA does not increase under salt stress; exactly, it may be due to that the ABA transfers from the roots to the shoots^74^. Conversely, our results showed that salt stress showed a greater increment in ACC, IPA, IAA and ABA in leaves of Chaoyouqianhao, whereas only the increased ABA content was observed in Huanghuazhan. Similar results were obtained by Zahedi et al., who reported that IAA and ABA contents increased in the presence of salinity in olive seedlings^75^.

ABA regulates the water state of plants by guarding cells, which contributes to overcome the adverse effects of salinity on growth, photosynthesis, and assimilates transport^3^. Foliar spraying ABA induced the stomatal closure and reduced the transpiration rate, which could be used as an anti-transpiration agent in production^76^. In the present study, ABA application strongly enhanced the photosynthetic parameters Pn, Gs and Tr in presence of salinity, it may be driven by an increased Gs, enhanced stomatal reopen and improved its function, thus improve Tr and maintain the stable state of Pn under salt stress; on the other hand, it may be that ABA effectively improves chlorophyll content and promotes the potential activity of PS II (Fv/Fo), thus enhancing plant photosynthesis^77^. Similar results were observed in lettuce^78^. Additionally, application of ABA stimulates the antioxidant system to protect salt toxicity from exaggerated photosynthetic electron transport in plants to enhance carbon assimilation^79^.

One of the mechanisms underlying plant salinity tolerance is the selective absorption and accumulation of inorganic ions, mainly Na^+^, K^+^, and Cl^−^^80^. It was demonstrated that ABA was a high efficient plant growth regulator in decreasing Na^+^ and Cl^−^ contents and Na^+^/K^+^ ratio, increasing K^+^ and Ca^2+^ concentrations^81^. In the present study, ABA significantly reduced Na^+^ contents in leaves of the two rice cultivars as well as in roots of Chaoyouqianhao, implied that ABA reduced transpiration by inhibiting K^+^ efflux channel, kept stomatal closure and resulted in a reduction in ion absorption in rices, thus a reduction in Na^+^ absorption was observed. On the other hand, Ca^2+^ as a secondary messenger initiates the stress signal transduction, leading to salinity adaptation. Previous study have showed that exogenous calcium reduced the effect of NaCl presumably by facilitating higher K^+^/Na^+^ selectivity^82^, which was well in line with our study, showing that a significant increase in Ca^2+^ in roots and leaves. In addition, it was found that Mg^2+^ increased under ABA-mediated salt stress, which may contribute to the enhancement of vacuole growth in leaves and roots, thereby isolating Na^+^ into vacuoles and limiting the accumulation of Na^+^ in shoot^83^.

Many studies have demonstrated that ABA could increase the antioxidant enzymes activity and alleviate the stress-triggered oxidative damage to plants^15,21,84^. However, the data that support this hypothesis remain inconsistent. For example, application of ABA in *kiwi fruit* under drought stress significantly improved the activity of POD, CAT, SOD, and APX^70^, whereas some results shown that ABA decreased CAT activity but significantly increased the activity of SOD and POD^85^. The present study displayed that ABA partly or completely increased the activity of CAT, APX, POD and SOD in roots, which depended on salt treatment time, suggesting that exogenous ABA upregulated antioxidant enzyme activity, thereby eliminating the ROS over-accumulation and protecting the cell membrane from oxidative damage. Conversely, these enzymes exhibited decreased trend in leaves of Huanghuazhan at 6 or 8 days after salt stress mediated by ABA. It may be that SOD is involved in elimination a part of ROS, resulting in reduced ROS generation and with that CAT,APX,POD activity decreased, which may also be due to the irreversible damage of plants under salt stress, caused the structure of enzyme protein damaged and resulting in decreased the enzyme activity. As a key enzyme of phenylpropanoids metabolism in plants, PAL plays an important role in abiotic stress. The increased activity under ABA treatment is presumably due to the up-regulation of PAL gene expression, thus improve the ability of stress resistance and promote the seedlings to adapt to the salt stress environment. However, the regulation of PAL gene in ABA-mediated salt stress pathway needs further study.

Previous study showed that exogenous application of GSH and AsA lead to reduced ROS accumulation and increased salt tolerance^86,87^. In our study, ABA strongly improved the level of AsA and PAL in the two rice seedlings, particularly in roots. As a key enzyme of phenylpropanoids metabolism in plants, PAL plays an important role in abiotic stress. The increased activity under ABA treatment is presumably due to the up-regulation of PAL gene expression, which regulates the synthesis and accumulation of flavonoid^88^, thus improve the ability of stress resistance and promote the seedlings to adapt to the salt stress environment. However, the regulation of PAL gene in ABA-mediated salt stress pathway needs further study. Notably, our results showed that ABA significantly reduced the level of GSH in roots at 6 or 8 days after salt stress in the two rice varieties, which consistent with previous reports^9,16^. Liting et al. pointed out that the differences of AsA and GSH in root and leaf tissues may be related to different functions, growth environment, and sensitivity of roots and leaves to drought stress stimulation and ABA^14^. Thus, we implied that the mechanism of ABA is different in the root and leaf tissues of rice seedlings.

Plant hormones are key endogenous chemical signals that plants coordinate plant growth and development under both optimal conditions and environmental stress^89^. Liu et al. pointed out that ABA significantly increased endogenous hormone contents such as ABA, GA, IAA and ZT^30^. Similar to our study, showing that ABA application increased the contents of ACC, TZ, IPA, IAA and ABA in Chaoyouqianhao (Table 4). Particularly, ACC content exhibited a strong increase (212.00%) when compared with salinity treatment; notably, it showed a stronger increase (317.80%) in Huanghuazhan. As a direct biosynthetic precursors of ethylene, ACC play an equal essential role in response to salt tolerance in rice, which has been extensively studied. Namely, promoting ethylene biosynthesis and signaling transduction can improve salt tolerance in plants^90^. Moreover, CTK belong to a class of plant growth substances (plant hormones) which includes IPA and TZ, regulating diverse events in plant growth and development. Results in our study showed that exogenous ABA increased the level of IPA and TZ under salt stress, especially in Huanghuazhan, which contributes to synthesis more CTK and thus regulated salt stress tolerance. Last but not least, the increased ABA contents mediated by ABA-stimulated under salt stress further regulated ROS scavenging, ion homeostasis, and stomatal closure^91^.

**Figure 10:**
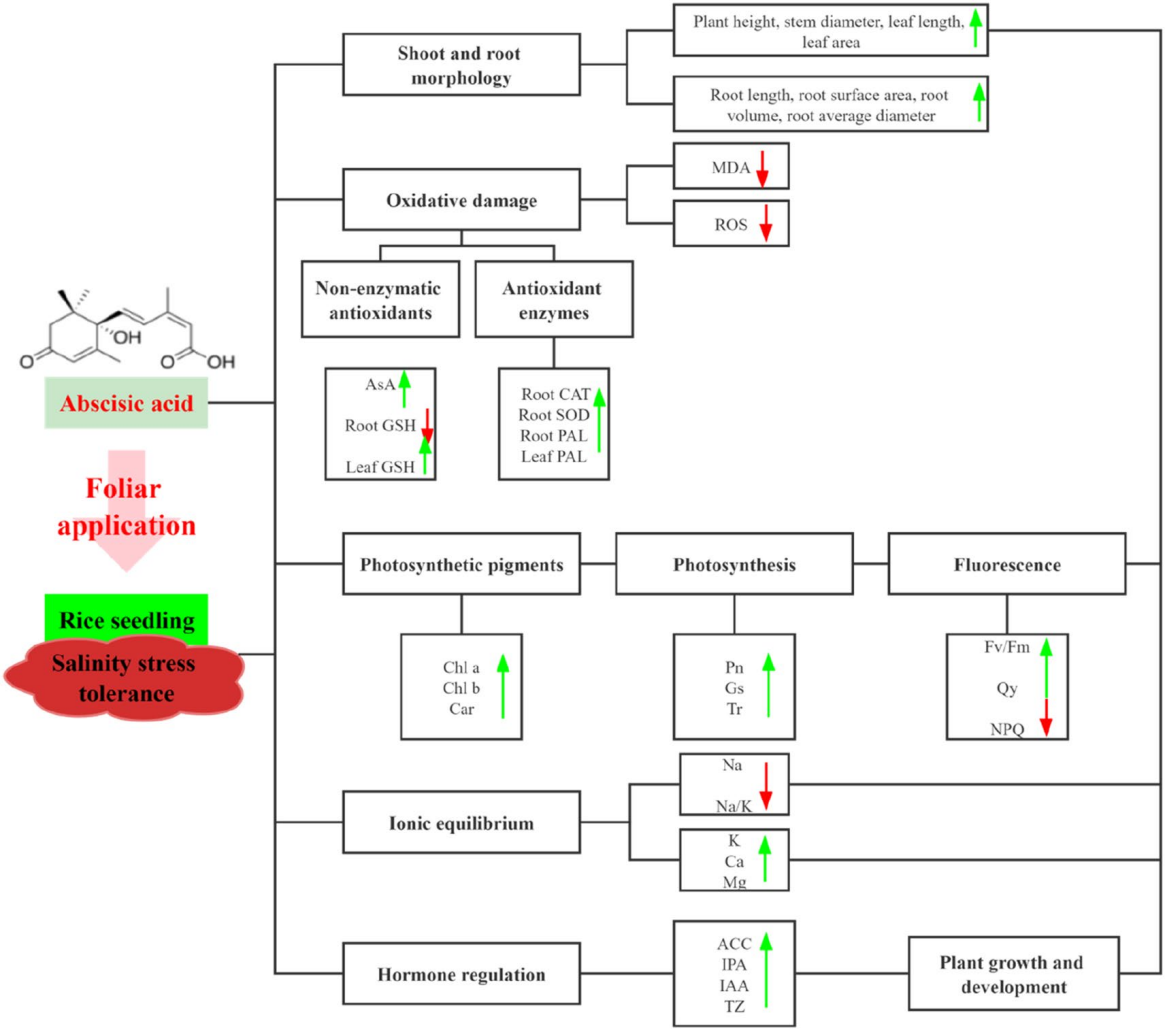
The pattern of ABA induced growth and development of rice seedling under salt stress. The up arrow and the down arrow indicate the increase and decrease of each index, respectively.

In conclusion, the present study reveals the physiological mechanisms underlying ABA-mediated the mitigation of salinity stress to plants. We focused on the response of root and leaf tissues to salt stress in two different rice cultivars and the alleviation effect of ABA application on salinity toxicity. Salt stress caused a significant reduction in plant morphological indexes and great changes in physiological characteristic of two rice cultivars. Foliar application of ABA mitigated the inhibitory effect caused by salinity on plant growth by improving photosynthesis, modifying the antioxidant enzymes (CAT, APX, POD, PAL) and non-antioxidant enzymes (AsA and GSH) as well as reducing oxidative damage, ultimately maintenance of ionic (low Na + and high K^+^/Na^+^) balance and salinity tolerance. Our study simultaneously demonstrated that the beneficial response and adaptation to salt stress requires the integration and coordination of multiple phytohormones. Further studies are needed to investigate the molecular mechanisms of ABA-mediated mitigation of salinity toxicity to plants. (A pattern diagram (Fig. 10) was added in Attachments).

## Materials and methods

### Plant materials and growth conditions

Seeds of two rice (*Oryza Sativa* L.) varieties, one inbred rice ‘Huanghuazhan’ (Z) and the other hybrid rice ‘Chaoyouqianhao’ (Q) were obtained from Guangdong Tianhong Seed Company Limited, Zhanjiang. Seeds were surface sterilized with a 2.5% sodium hypochlorite solution for 15 min, followed by rinsing three times with distilled water, and then the seeds were germinated with distilled water in the dark for 2 days at 30 ℃. After that 75 uniformly germinated seeds were used as experimental materials for growing per plastic pot (19.5 × 14.5 × 17.5 cm) containing 3.0 kg of substrates (Latosol: sand = 3:1, v/v). In the present test, a factorial experiment based on completely randomized design was carried out. Briefly, 30 pots were used per plot (represent one of eight treatments, treatments were list as following), and therefore a total of 240 pots were used in the study.

When the rice seedlings had naturally grown to the 2 leaf/1 heart stage (8 days after planting), seedlings were foliar spraying with 5 mg L^−1^ S-ABA (provided by Sichuan Lomon Fusheng Technology Co., Ltd), using a small hand-held sprayer, each pot was added with 20 mL liquid. Specially, the concentration of ABA was chosen according to previous pot experiment study (Fig. 1), then 25 mM NaCl (w/w) solution was added to each pot after 24 h, and the other 25 mM NaCl solution was added on the 3^rd^ day of ABA treatment to got the desired salinity level (50 mM). The following applications of S-ABA and NaCl were applied to each experimental pot: (i) (Q0 or Z0): 0 mg L^−1^ S-ABA + 0 mM NaCl, (ii) (Q1 or Z1): 5 mg L^−1^ S-ABA + 0 mM NaCl, (iii) (Q2 or Z2): 0 mg L^−1^ S-ABA + 50 mM NaCl, (iv) (Q3 or Z3): 5 mg L^−1^ S-ABA + 50 mM NaCl. The plant samples were harvested at 2, 4, 6, 8, 10 days after salt stress for morphological and physiological analysis.

### Observations

#### Growth response measurements

Samples of roots and shoots were collected at the treatment intervals. Plant height, stem base width, leaf area, leaf length, shoot dry weight and root dry weight were measured. For determining dry weight, shoots and roots were separately placed in paper bags, and then placed in an oven at 80 ℃ for 48 h. For determining RL, RSA, RV and RAD, root system scanner (Epson Perfection V800 Photo (Epson Indonesia Inc.)) and analysis software WinRHIZO were used. All the values for each parameter were mean of at least four independent replicates. Root to shoot ratio was calculated according to the following formula:

$${\text{Root}}\;{\text{ to}}\;{\text{ shoot}}\;{\text{ ratio = Root }}\;{\text{dry }}\;{\text{weight/Shoot }}\;{\text{dry}}\;{\text{ weight}}$$

### Measurement of photosynthetic pigments

The concentration of Chl was measured according to the protocol described by Arnon^92^ with a slight modification. Chl a, Chl b and Car concentration were measured spectrophotometrically at 665, 649 and 470 nm, respectively.

$$\begin{aligned} & {\text{Chl}}\;{\text{ a (mg}}\;{\text{ g}}^{{ - {1}}} {) = 13}{\text{.95}}\;{\text{ A665}} - {6}{\text{.88}}\;{\text{A649}} \\ & {\text{Chl}}\;{\text{ b (mg}}\;{\text{ g}}^{{ - {1}}} {) = 24}{\text{.96 }}\;{\text{A649}} - {7}{\text{.32}}\;{\text{A665}} \\ & {\text{Total}}\;{\text{ Chl }}\;{\text{(mg}}\;{\text{ g}}^{{ - {1}}} {\text{) = Chl }}\;{\text{a + Chl}}\;{\text{ b}}{.} \\ & {\text{Car}}\;{\text{ (mg}}\;{\text{ g}}^{{ - {1}}} {) = (1000}\;{\text{ A470}} - {2}{\text{.05}}\;{\text{Chl}}\;{\text{a}} - {114}{\text{.8}}\;{\text{Chl}}\;{\text{b)/245}} \\ \end{aligned}$$

### Measurement of photosynthetic parameters

After 2, 4, 6, 8 and 10 days of salt stress, P_n_, G_s_, C_i_ and T_r_ were measured by using portable photosynthesis system LI-6400 (LI-COR, Inc., USA). The measurements were made between 14:00 and 16:00 P.M. The CO_2_ concentration in the leaf chamber was 400 μmol·mol^−1^, the air velocity was 500 μmol·s^−1^, the light intensity was 1000 μmol·m^−2^ ·s^−1^, the leaf temperature was 32 ± 1℃, and the relative air humidity was between 70 and 80%.

### Measurement of chlorophyll fluorescence parameters

Fluorescence parameters were measured using portable Chlorophyll Fluorometer (FluorPen FP110, Czech) in dark condition at night at 2, 4, 6, 8, 10 days after salt stress, including Fv/Fm, Fv/Fo, Fm/Fo, NPQ, Qp, Qy and Rfd.

### Measurement of ions contents

After 8 days of salt stress, Na^+^, K^+^, Mg^2+^ and Ca^2+^ contents were determined in 150 mg dry mass (DW) of leaves and roots. Samples were digested in 6 ml of HNO_3_/HClO_4_ (4:1, v/v), and supernatants after clarifying the digestate were analyzed. Na^+^, Mg^2+^ and Ca^2+^ contents were measured by Inductively Coupled Plasma Emission Spectrometry (Prodigy XP, LEEMAN, Inc., USA) and the K^+^ content was measured by flame photometer (Sherwood M410, Inc., UK).

### Analysis of lipid peroxidation

Lipid peroxidation was measured in terms of MDA level using the TBA method as described by Cakmak and Horst^93^. Absorbance of the supernatant was measured at 450, 532 and 600 nm. The MDA-TBA complex was quantified using the extinction coefficient as 155 mM^−1^ cm^−1^. The determination of H_2_O_2_ concentration was measured according to previous study^94^.

### Measurement of antioxidant enzyme activity

0.5 g of fresh root and leaf tissues were ground into fine powder under liquid nitrogen, and then 10 mL of precooled phosphate buffer (50 mM, pH 7.8) containing 1.0% (w/v) PVP was added and homogenized. Mixture was centrifuged for 40 min at 7000 × g and 4 ℃. The obtained supernatant was used for enzyme assays as the crude enzyme preparation. The CAT^95^, APX^96^ and POD^97^ activity were assayed as described by Ekinci et al., Nakano and Asada, Chance and Machly, respectively. Since the addition of H_2_O_2_, the absorbance changes were monitored for 120 s at 240 nm, 290 nm and 470 nm for assayed the CAT, APX and POD activities, respectively. The SOD activity was assayed as described by Spychalla and Desborough^98^. One unit of SOD activity was defined as the amount of enzyme needed to cause 50% inhibition of the photoreduction of NBT as monitored at 560 nm.

### Measurement of antioxidant molecules

The AsA content was measured according to the method of Kampfenkel^99^. The absorbance value was read at 534 nm. The level of GSH content was assayed as described by Tyburski and Tretyn^100^ with a slight modification. The reaction mixture contained 200 μL supernatant, 2.6 mL of acetic acid buffer (0.2 M, pH 5.6) and 200 μL of 5,5-dithio-bis-(2-nitrobenzoic acid), mixture was kept at 30 ℃ for 10 min. The absorbance value of GSH was read at 412 nm. For the determination of PAL, the reaction mixture consisted of 1.9 mL of 10 mM L-phenylalanine and 100 μL supernatant that was reacted at 30 ℃ for 30 min in the dark. The absorbance value was read at 290 nm^101^.

### Measurement of soluble protein content

The level of soluble protein was measured using the Coomassie brilliant blue method^102^. The absorbance of the supernatant was measured at 595 nm after 2 min reaction, and then figure out the protein content in the sample according to the standard curve with bovine serum albumin.

### Measurement of endogenous hormone content

After 8 days of salt stress, endogenous contents of ACC, TZ, IPA, IAA and ABA in leaves of Chaoyouqianhao (Q) and Huanghuazhan (Z) were determined by UHPLC-MRM-MS/MS following of previous protocols^103^.

### Statistical analysis

The data analysis and the graphical presentation using the Microsoft Excel 2019 and Origin 2019. A factorial experiment based on completely randomized design was carried out and statistical significance of the means for at least four replications were compared by Duncan's multiple range tests, at the five percent probability level using SPSS software version 19.0.

## Abbreviations

RAD: Root average diameter
RL: Root length
RSA: Root surface area
RV: Root total volume
P_n_: Net photosynthesis rate
C_i_: Intercellular CO_2_ concentration
G_s_: Stomatal conductance
T_r_: Transpiration rate
Chl: Chlorophyll
Fv/Fm: Maximal photochemical efficiency of PS II
Fm/F0: Electron transfer case of PS II
Qy: Effective photochemical efficiency of PS II
Qp: Photochemical quenching coefficient
NPQ: Non-photochemical quenching coefficient
Rfd: Fluorescence decrease ratio
SOD: Superoxide dismutase
POD: Peroxidase
CAT: Catalase
APX: Ascorbate peroxidase
PAL: Phenylalnine ammonialyase
AsA: Ascorbic acid
GSH: Glutathione
ROS: Reactive oxygen species
O_2_^−^: Superoxide
OH^−^: Hydroxyl free radicals
1O_2_: Singlet oxygen
H_2_O_2_: Hydrogen peroxide
MDA: Malondialdehyde
ABA: Abscisic acid
IAA: Indole-3-acetic acid
CTK: Cytokinins
ZT: Zeatin
Tz: Trans-Zeatin
ACC: 1-Aminocyclopropanecarboxylic acid
IPA: N6-isopentenyladenosine
GA: Gibberellin
JA: Jasmonic acid
SA: Salicylic acid
PVP: Polyvinylpyrrolidone
TBA: Thiobarbituric acid
